# Topological organization and dynamic regulation of human tRNA genes during macrophage differentiation

**DOI:** 10.1186/s13059-017-1310-3

**Published:** 2017-09-20

**Authors:** Kevin Van Bortle, Douglas H. Phanstiel, Michael P. Snyder

**Affiliations:** 1 Department of Genetics, Stanford University, Stanford, CA 94305 USA; 2 Department of Cell Biology and Physiology, University of North Carolina, Chapel Hill, NC 27599 USA; 3 Thurston Arthritis Research Center and Department of Cell Biology and Physiology, University of North Carolina, Chapel Hill, NC 27599 USA

**Keywords:** tDNA, tRNAome, Hi-C, Biotin-capture, CTCF, Topologically associating domains, TFIIIC

## Abstract

**Background:**

The human genome is hierarchically organized into local and long-range structures that help shape cell-type-specific transcription patterns. Transfer RNA (tRNA) genes (tDNAs), which are transcribed by RNA polymerase III (RNAPIII) and encode RNA molecules responsible for translation, are dispersed throughout the genome and, in many cases, linearly organized into genomic clusters with other tDNAs. Whether the location and three-dimensional organization of tDNAs contribute to the activity of these genes has remained difficult to address, due in part to unique challenges related to tRNA sequencing. We therefore devised integrated tDNA expression profiling, a method that combines RNAPIII mapping with biotin-capture of nascent tRNAs. We apply this method to the study of dynamic tRNA gene regulation during macrophage development and further integrate these data with high-resolution maps of 3D chromatin structure.

**Results:**

Integrated tDNA expression profiling reveals domain-level and loop-based organization of tRNA gene transcription during cellular differentiation. tRNA genes connected by DNA loops, which are proximal to CTCF binding sites and expressed at elevated levels compared to non-loop tDNAs, change coordinately with tDNAs and protein-coding genes at distal ends of interactions mapped by in situ Hi-C. We find that downregulated tRNA genes are specifically marked by enhanced promoter-proximal binding of MAF1, a transcriptional repressor of RNAPIII activity, altogether revealing multiple levels of tDNA regulation during cellular differentiation.

**Conclusions:**

We present evidence of both local and coordinated long-range regulation of human tDNA expression, suggesting the location and organization of tRNA genes contribute to dynamic tDNA activity during macrophage development.

**Electronic supplementary material:**

The online version of this article (doi:10.1186/s13059-017-1310-3) contains supplementary material, which is available to authorized users.

## Background

The role of transfer RNAs (tRNAs) in deciphering the genetic code is universal to cell biology. The trinucleotide anticodon sequence of each tRNA-type decodes specific codons employed by messenger RNAs (mRNAs). Overall, the number of genes encoding each tRNA-type and the relative cellular abundance of each tRNA-type have been shown to correlate with the frequency of codon usage in species-specific and tissue-specific contexts, respectively [1–3]. In eukaryotes, changes in tRNA abundance have been reported across proliferative and senescent cell types and in response to specific perturbations, such as exposure to oxidation and alkylation-related stress [3–5]. Several important extra-translational functions for tRNA and tRNA-derived fragments have also become apparent, such as interfering with transposon reactivation and antagonizing the stability of oncogenic transcripts in breast cancer cells [6–8]. Thus, adjusting the level of cellular tRNA molecules, through both transcriptional and post-transcriptional mechanisms, may be important for modulating translation and potential ancillary activities.

Deciphering the mechanisms by which nascent tRNA levels are dynamically regulated, however, remains difficult to address, due in part to the unique challenges related to tRNA sequencing and alignment, as well as the unique complexity of tRNA biology [9]. In regard to sequencing, cellular tRNAs are heavily modified and consequently difficult to reverse transcribe during library preparation. In recent years, studies have tackled specific challenges associated with tRNA sequencing, or utilized alternate means, such as mapping RNA polymerase III as readout for tDNA expression [10–12]. The use of a dealkylating enzyme, ALKB, improves the fraction of full-length tRNA reads by demethylating sites that block reverse transcription [13–16]. However, sequencing of cellular tRNA levels alone provides little information about the transcriptional activity of tRNA genes, as nascent tRNAs undergo a complex maturation process [17]. Mapping of RNA polymerase III, meanwhile, represents an imperfect measure of tRNA gene activity that does not directly assay the level of nascently transcribed RNA. To this end, biotin-capture based genomic run-on experiments, such as BioGRO and precision nuclear run-on sequencing (PRO-seq), allow quantitative transcriptional profiling and mapping of RNA polymerases [18–21]. Thus, leveraging both RNA polymerase III occupancy with biotin-capture of nascent, demethylated tRNAs may provide a more accurate measure of tRNA gene expression in growing cells.

Interaction-based studies profiling the structure of eukaryotic chromosomes have identified highly self-interacting topological domains, a unit of three-dimensional (3D) organization that divides the genome into local neighborhoods of similar gene activity and restricts the ability of enhancers to influence non-target genes [22–29]. Recent studies mapping global interaction frequencies by in situ high-throughput chromosome conformation capture (in situ Hi-C) have further improved the resolution of physical domain identification and suggest that these contact domains are largely stable across cell types [30–32]. These short-range structures are often established within loops connected by inward oriented CTCF binding sites, an architectural protein originally described by its ability to function as an insulator, and by the cohesin complex and factors that control its association with DNA [30, 33–40]. tRNA genes, which are also enriched at the boundaries of topological domains and, in certain contexts, have been shown to function as insulator elements in the classical sense, have also been reported to play a role in the organization of eukaryotic chromosomes [28, 41–44]. However, to what degree tRNA genes are involved in long-range interactions in humans, and whether the 3D organization of tRNA genes contributes to the activity of these genes themselves remains unknown.

We have recently profiled the 3D organization and long-range interactome of human THP-1 monocytes and THP-1-derived macrophages through deeply sequenced in situ Hi-C experiments. High-resolution mapping of DNA loops identified both static and dynamic loop-based regulation of key macrophage genes during cellular differentiation [45]. THP-1 monocytes were differentiated into macrophages by treating with phorbol myristate acetate (PMA), which induces significant changes in cellular morphology and expression of cell surface markers characteristic of macrophages [46–48]. THP-1 cells, which typically grow in suspension, become adherent within 72 h post PMA treatment, providing a straightforward method for isolating relatively pure populations of non-differentiated monocytes and THP-1-derived macrophages [49]. Isolation of homogeneous cell populations is particularly appealing for the study of tRNA gene dynamics during cellular differentiation, as tDNAs, which are essential for biosynthesis, are likely to exhibit comparatively subtle changes in transcription.

Here we present integrated tDNA expression profiling, a method that combines RNAPIII occupancy mapping with biotin-capture of nascent, demethylated tRNAs. We apply this method to the study of dynamic tRNA gene regulation during macrophage development and further integrate these data with our recently described maps of 3D chromatin structures in the same cell types. Integrated tDNA expression profiling reveals domain-level and loop-based organization of transcription during cellular differentiation, as well as dynamic transcription factor (TF) binding coincident with changes in tDNA transcription, altogether revealing novel features of tRNA gene regulation.

## Results

### Integrated tDNA expression profiling in THP-1 monocytes

**Fig. 1:**
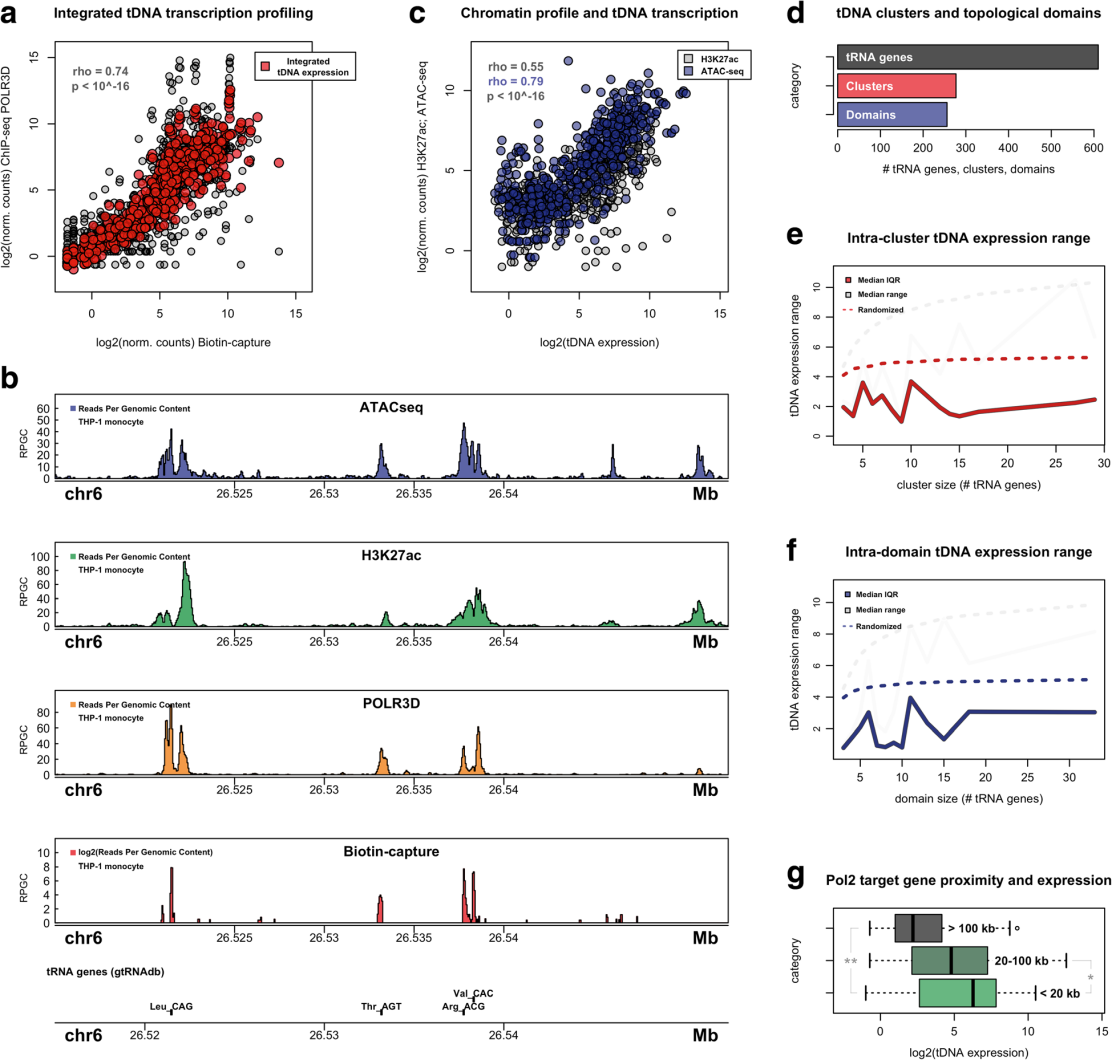
Integrated tDNA expression and chromatin profiling in THP-1 monocytes. **a** Correlation between tRNA gene expression as measured by biotin-capture of nascent, demethylated tRNAs and by RNA polymerase III occupancy mapping by ChIP-seq (*black*; Spearman’s rank correlation coefficient = 0.74; *p* < 10^-16). Integrated tDNA expression profile (*red*) utilizes the mean normalized count for each tRNA gene. **b** Example signal track representation of the chromatin accessibility (ATAC-seq, *blue*), active histone signature H3K27 acetylation (ChIP-seq, *green*), RNA polymerase III occupancy (ChIP-seq, *orange*), and nascently transcribed RNA (Biotin-capture, *red*) at a tDNA cluster located on chromosome 6. *RPGC* mean normalized reads per genomic content. **c** Correlation between integrated tDNA expression profile with H3K27ac ChIP-seq levels surrounding tRNA genes (*black*; Spearman’s rank correlation coefficient = 0.55; *p* < 10^-16) and with chromatin accessibility at tRNA genes as measured by ATAC-seq (*blue*; Spearman’s rank correlation coefficient = 0.79; *p* < 10^-16). **d** Number of tRNA genes, tDNA clusters, and physical contact domains containing tRNA genes in human THP-1 cells. **e** Median intra-cluster tDNA expression range (*gray*) and interquartile range (IQR; red) as a function of tDNA cluster size (number of tRNA genes present within each cluster). *Dotted lines* represent median range and IQR for randomly shuffled tRNA genes within tDNA clusters (100,000 permutations). **f** Median intra-domain tDNA expression range (*gray*) and IQR (*blue*) as a function of tDNA contact domain size (number of tRNA genes located within each contact domain). *Dotted lines* represent median range and IQR for randomly shuffled tRNA genes within contact domains (100,000 permutations). In the case of overlapping contact domains, tRNA genes were assigned to the single smallest resident domain. **g** Distribution of integrated tDNA expression values segregated by proximity to nearest RNA polymerase II-transcribed gene (>100 Kb; n = 81; 20–100 Kb; n = 232; < 20 Kb; n = 295; **p = 1.18^-10, *p = 0.02, Wilcoxon rank-sum test)

To directly measure tRNA gene transcription in human THP-1 cells, we combined a previously described tRNA demethylation strategy with a biotin-capture based method for isolating nascently transcribed RNAs. With this approach, the complex cytoplasmic mixture of intermediate, mature aminoacyl-tRNA and tRNA fragments that are captured by conventional RNA-sequencing (RNA-seq) methods do not preclude our ability to specifically query nascent tRNA levels. Mapping of tRNA fragments, nevertheless, remains problematic due to the multi-copy nature of tRNA genes; multiple alignment and analysis strategies have been proposed [50, 51]. For tDNA transcription profiling, we chose to map nascent RNA reads to the entire human genome space to avoid false positives arising from sequence reads that are unrelated to tRNAs [50]. Additionally, the presence of non-templated “CCA” at the 3’ terminus of mature tRNAs were not considered within the context of tRNA gene transcription. As an independent assay, we mapped the occupancy of RNA polymerase III genome-wide by chromatin immunoprecipitation sequencing (ChIP-seq), targeting the POLR3D (RPC4) subunit as previously described [21]. Overall, biotin-capture and RNA polymerase III mapping experiments show strong correlation in estimated tDNA expression values for individual genes (Fig. 1a). Because tRNA alignment is an imperfect process and RNA polymerase III mapping indirectly defines tDNA transcription, we chose to integrate both measures of tDNA expression for all downstream analyses (see “Methods”). Importantly, inspection of integrated tDNA expression estimates demonstrates high correlation and reproducibility across biological replicates (Additional file 1: Figure S1a, Pearson correlation coefficient = 0.978; *p* < 10^-16).

In total, we estimate the expression for 610 tRNA genes currently present in the human genomic tRNA database (gtRNAdb, hg19) [52, 53]. Integrated tDNA expression levels show a bimodal distribution consistent with previous reports suggesting that nearly half of all tRNA genes are not occupied by RNA polymerase III, resulting in little or no transcription (Additional file 1: Figure S1b) [54–56]. To better characterize the environmental context of tRNA gene transcription, we further profiled chromatin accessibility at tRNA genes using an assay for transposase accessible chromatin (ATAC-seq), as well as the level of histone H3K27 acetylation (H3K27ac), a histone modification positively associated with transcription levels at both RNA polymerase II and RNA polymerase III genes (Fig. 1b) [3, 57–60]. Correlation analysis reveals statistically significant relationships for both H3K27ac and chromatin accessibility with integrated tDNA expression levels at individual tRNA genes (Fig. 1c). The strong relationship observed between tDNA transcription and ATAC-seq (Spearman’s rank correlation coefficient = 0.79, *p* < 10^-16) suggests that DNA accessibility may uniquely capture the activity of short, non-coding tRNA genes, which are depleted of nucleosomes within the gene body and promoter regions [61]. Together, measures of tDNA accessibility and H3K27ac appropriately reflect the transcriptional activity of human tRNA genes and indicate that integrated tDNA expression profiling accurately captures nascent tRNA gene transcription levels.

### Domain-level organization of human tRNA genes

We next considered whether the linear arrangement of tRNA genes into clusters and physical contact domains plays a role in the organization of tRNA gene transcription. Contact domains were annotated in THP-1 cells using the arrowhead algorithm as previously reported [30], which in total identifies more than 12,000 domains with enhanced contact frequency [45]. Altogether, we identify 256 physical contact domains containing one or more tRNA genes, with an average size of 2.3 resident tRNA genes per domain and a maximum size of 33 tRNA genes per domain (Fig. 1d). However, domain mapping alone does not encompass the entire human genome and thus we performed parallel analysis of tDNA clustering to comprehensively compare the expression of all human tRNA genes (Additional file 1: Figure S1c). Clusters were analogously defined as regions of DNA with one or more tDNAs, using a maximum tDNA-tDNA distance threshold of 20 Kb. In total, we identify 277 individual tDNA clusters with an average size of 2.2 tRNA genes per cluster and a maximum size of 29 tRNA genes per cluster (Fig. 1d).

We find that the median expression level for tRNA genes increases with the number of neighboring tDNAs, leveling off at a cluster size of approximately four tRNA genes (Additional file 1: Figure S1d). Inspection of individual multi-tDNA clusters and physical contact domains further suggests that proximal tRNA genes may be expressed at similar levels (Additional file 1: Figure S1e, f). To systematically determine whether tRNA genes present in the same cluster or domain do share similar gene activity, we compared the range and interquartile range (IQR) of tDNA transcription with a model in which tRNA genes are randomly assigned with respect to cluster and domain occupancy. Indeed, we find significantly lower spread in tRNA gene expression values across all cluster and domain sizes (Fig. 1e, f, Additional file 1: Figure S1g), suggesting cluster- and domain-level organization of human tDNAs group genes with similar transcriptional activity. We further find that tRNA gene expression positively associates with the proximity of tDNAs to genes transcribed by RNA polymerase II (Fig. 1g). These results agree with previous observations indicating correlative proximity and activity between neighboring RNA polymerase II and RNA polymerase III genes [60, 62, 63], and altogether argue that the surrounding context is an important aspect of tRNA gene transcription.

### tDNA organization, transcription, and codon usage in THP-1 cells

**Fig. 2:**
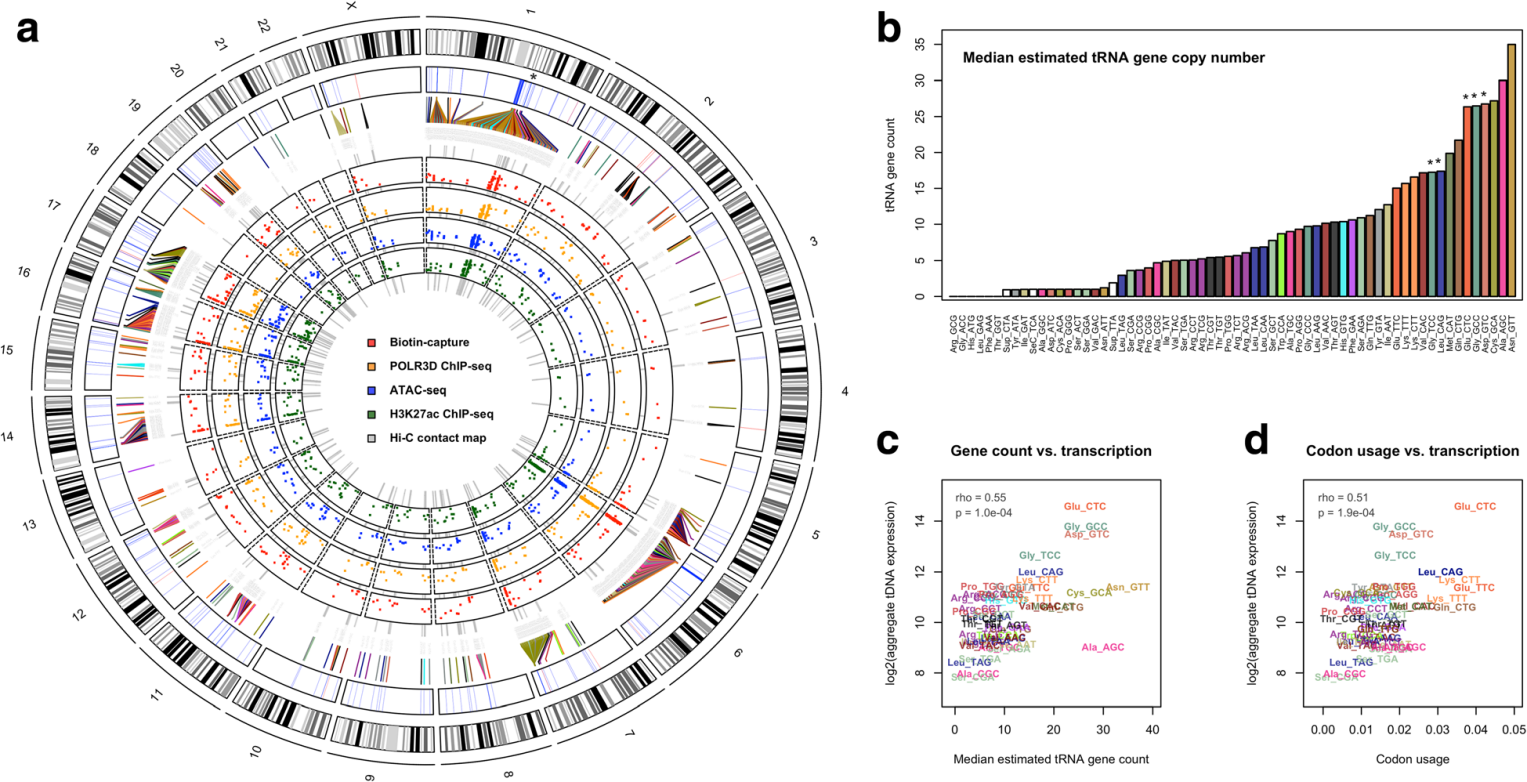
Organization and transcription of multicopy tRNA genes in humans. **a** Circular visualization of human tRNA gene coordinates across human chromosomes. Track descriptions from outermost moving inward: (1) Individual chromosome ideograms and cytogenetic band positions. (2) Location of tRNA genes (*blue*) and nuclear-encoded mitochondrial nmt-tRNA genes (*red*). *Asterisk represents approximate location of variable number tandem repeat (VNTR) on chromosome 1. (3) tRNA genes labeled by tRNA anticodon family and colored by amino acid isoacceptor family (note: not all tDNAs are directly labeled due to size and legibility constraints). (4) Biotin-capture based assay measuring nascent demethylated tRNAs (*red*). (5) ChIP-seq experiments mapping RNA polymerase III occupancy on tRNA genes (*orange*). (6) ATAC-seq experiments measuring chromatin accessibility at tRNA genes (*blue*). (7) ChIP-seq experiments capturing H3K27 acetylation levels at tRNA genes (*green*). (8) Link-plot of DNA loops identified by in situ Hi-C experiments proximal to tRNA genes (*gray*). **b** Median estimated human tRNA gene count for each anticodon tRNA family, colored by amino acid, as determined using a read-depth approach over several deeply sequenced whole-genome datasets from the 1000 Genomes Project. *Asterisks represent tRNA-types located on VNTR that show significant variation across sampled individuals. **c** Correlation between integrated tDNA expression, collapsed by anticodon tRNA family, with multi-copy tRNA gene count (Spearman’s rank correlation coefficient = 0.55; *p* = 1.0e-04). **d** Correlation between integrated tDNA expression, collapsed by anticodon tRNA family, and the frequency of cognate codon usage in the THP-1 transcriptome for multicopy tRNA genes (Spearman’s rank correlation coefficient = 0.51; *p* = 1.9e-04)

Recent studies suggest that significant copy number variation may exist for tRNA genes across the human population [64]. We therefore performed a read-depth approach for estimating tRNA gene copy number in several deeply and moderately sequenced whole-genome datasets available through the 1000 Genomes Project [65]. Of note, we observe a high degree of variation across individuals at specific tDNAs, particularly at a set of genes encoding five distinct tRNA anticodon families that are located within a recently described variable number tandem repeat (VNTR) on chromosome 1 (Fig. 2a, b; asterisks, Additional file 1: Figure S2a, b) [66]. As expected, tDNA copy number estimates scale appropriately with VNTR number and, overall, our median tRNA gene copy number estimates show improved correlation between gene count and aggregate nascent tRNA levels for multi-tDNAs in THP-1 monocytes (Fig. 2c, Spearman’s rank correlation coefficient = 0.55; *p* = 0.0001, Additional file 1: Figure S2c, d).

We observe a similar correlation between aggregate tRNA levels and the codon usage of the THP-1 transcriptome (Fig. 2d), further serving to validate the accuracy and quality of our integrated tDNA expression profiles. Transcription of tRNA genes is particularly better adjusted towards codon usage than gene count with respect to high copy number tDNAs (Additional file 1: Figure S2e). For example, tRNA^Ala-AGC^ and tRNA^Asn-GTT^ represent two of the highest anticodon families in terms of estimated tRNA gene count, yet they decode moderately employed codons in THP-1 cells (Fig. 2c). The aggregate levels of nascent tRNA^Ala-AGC^ and tRNA^Asn-GTT^ are instead better adjusted to codon usage (Fig. 2d), suggesting transcription of human tRNA genes is regulated beyond gene count.

Visualization of tRNA gene coordinates with respect to tRNA-type illustrates the level of overlap between distinct anticodon families across all human chromosomes (Fig. 2a). Hierarchical clustering of tRNA families by overlap frequencies, that is the number of times genes encoding two distinct tRNA-types are located within the same tDNA cluster, reveals preferential proximity between specific pairs of anticodon tRNA families (Additional file 1: Figure S2f). The tDNAs located within the VNTR on chromosome 1, for example, exhibit strong overlap frequencies and together represent several of the highest expressed tRNA species in THP-1 monocytes (Fig. 2b–d). Genes encoding tRNA-types with strong overlap frequencies tend to segregate by tDNA expression levels, reaffirming the important relationship between tRNA gene organization and transcription (Additional file 1: Figure S2f).

### Dynamic transcription of tRNA genes during macrophage differentiation

**Fig. 3:**
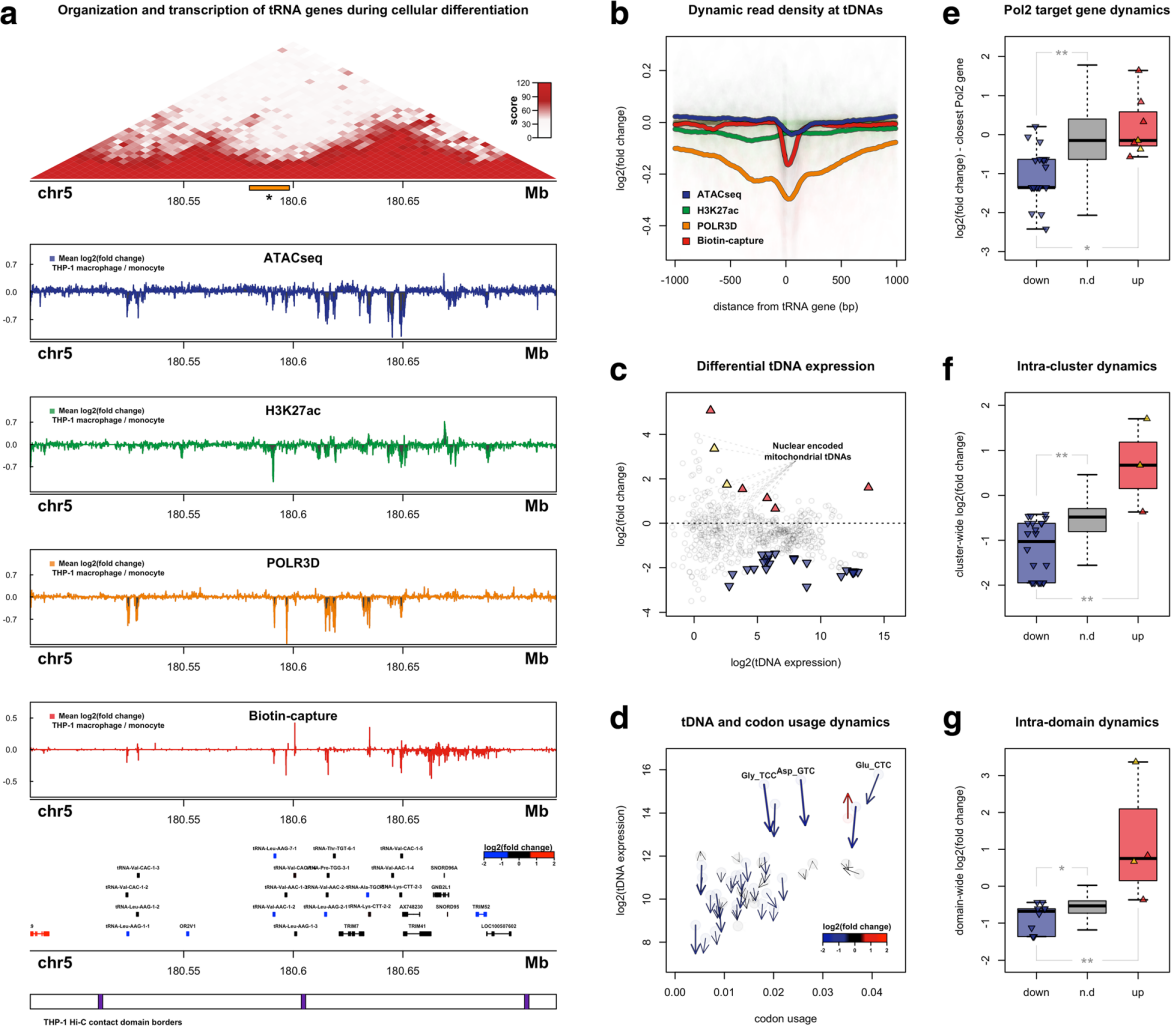
Dynamic domain-level regulation of tRNA gene transcription during macrophage differentiation. **a** Visualization of chromatin and transcriptional dynamics at an example tDNA locus on chromosome 5. *Top*: in situ Hi-C contact frequency matrix in THP-1 monocytes. *Asterisk represents long-range loop anchor region presented in Fig. 4c. *Middle*: mean log2(fold change) signal tracks for chromatin accessibility (ATAC-seq; *blue*), H3K27 acetylation (ChIP-seq; *green*), RNA polymerase III occupancy (ChIP-seq; *orange*), and nascent RNA (Biotin-capture RNA-seq; *red*) across two adjacent contact domains and neighboring tDNA clusters. *Bottom*: gene structure and physical contact domain border locations. log2(fold change) represents ± 72 h PMA treatment. Gene structure includes both tRNA genes and proximal RNAPII-transcribed genes. *Lower panel* depicts corresponding contact domain borders in THP-1 monocytes. **b** Mean log2(fold change) in normalized signal track read density for ATAC-seq, H3K27ac, POLR3D, and Biotin-capture RNA-seq across all human tRNA genes ± 1 Kb (−1000 upstream, +1000 downstream). **c** Mean log2(fold change) of integrated tDNA expression values across individual tRNA genes and mean log2(integrated tDNA expression) comparing THP-1 cells ± 72 h PMA treatment. *Triangles* represent individual tRNA genes that are upregulated (*red*) and downregulated (*blue*) at an FDR threshold of 0.15, including significant nmt-tDNAs (*gold*). **d** *Arrow plot* representation of aggregate nascent tRNA dynamics collapsed by tRNA-type vs. codon usage frequency (labeled by anticodon). *Arrow-head* represents log2(tDNA expression) and cognate codon usage after 72 h PMA treatment; *arrow-bottom* represents log2(tDNA expression) and cognate codon usage in untreated THP-1 monocytes. **e** log2(fold change) in closest RNAPII-transcribed genes for downregulated (*blue*), upregulated (*red*), and non-differential (n.d., *gray*) tRNA genes (*top* **p = 1.58^-5; *bottom* *p = 0.047, Wilcoxon rank-sum test). **f** Median cluster-wide log2(fold change) for tDNA clusters harboring downregulated (*blue*), upregulated (*red*), and non-differential (*gray*) tRNA genes (*top* **p = 0.0056, bottom **p = 6.41^-6, Wilcoxon rank-sum test). **g** Median contact domain-wide log2(fold change) for tDNA clusters harboring downregulated (*blue*), upregulated (*red*), and non-differential (*gray*) tRNA genes (*top* *p = 0.016; *bottom* **p = 0.0022, Wilcoxon rank-sum test)

The temporal dynamics of PMA-induced THP-1 differentiation include a distinct early response after 6 h, followed by a transition towards differentiation completion at 48–96 h, at which point THP-1 cells become adherent and express macrophage-related cell surface markers [48, 49]. We therefore profiled tDNA transcription, chromatin accessibility, and long-range interactions in adherent, THP-1-derived macrophages 72 h post PMA treatment (Fig. 3a). Comparison of tDNA expression before and after PMA treatment suggests that transcription of tRNA genes generally decreases during macrophage differentiation. This trend is consistently observed with respect to chromatin accessibility, RNA polymerase III occupancy, and nascent tRNA levels, both in terms of read density and normalized differential count-based analysis over all tRNA genes (Fig. 3b, Additional file 1: Figure S3a). Differential analysis of integrated tDNA expression profiles similarly identifies a bias of downregulation at most individual tRNA genes 72 h post PMA treatment (Fig. 3c). Nevertheless, transcription of several nuclear-encoded mitochondrial tRNA genes (nmt-tRNAs) increase in THP-1-derived macrophages (Fig. 3c, Additional file 1: Figure S3b–d). This finding is congruous with an observed increase in mitochondrial numbers during macrophage development and in PMA-stimulated THP-1 cells [48], suggesting a potential increase in the demand for mitochondrial protein synthesis.

The collective decrease in non-mitochondrial tDNA expression is consistent with previous comparisons of RNA polymerase III occupancy in human embryonic stem cells and induced pluripotent stem cells, which suggest that differentiation leads to a constricted RNAPIII repertoire [56]. We find that downregulation of nascent tRNA levels is most pronounced for the highest expressed tRNA-types, suggesting macrophage differentiation decreases the dynamic range of tRNA availability in THP-1 cells (Fig. 3c, d). This decrease in the most abundant tRNA anticodon families correlates with a decline in codon usage for several of the most frequently employed codons (Fig. 3d, Additional file 1: Figure S3h), suggesting a potentially coordinated decrease in the dynamic range for both tRNA supply and mRNA demand. Thus, we speculate that cell-type-specific tRNA levels may be adjusted in a manner that complements the dynamic range of codon usage rather than specific codon frequency optimization.

### tDNA dynamics mirror the surrounding transcriptional environment of tRNA genes

Given the relationship between tDNA activity and proximity to RNA polymerase II genes, we next asked whether changes in tRNA gene expression coincide with the transcriptional environment surrounding differential tRNA genes. Indeed, tDNAs that decrease or increase significantly in THP-1-derived macrophages show enrichment for similar changes in nearby protein-coding genes (Fig. 3e, Additional file 1: Figure S3e). Beyond RNAPII transcribed genes, tDNAs also share similar transcriptional dynamics with tRNA genes that reside within the same cluster or topological domain. When tDNA transcription is compared with the median fold change across tDNA clusters and domains, increasing and decreasing tRNA genes again are biased towards genes the behave similarly, both for tDNA clusters (Fig. 3f) and for physical contact domains containing tRNA genes (Fig. 3g, Additional file 1: Figure S3f, g). Visual inspection of transcriptional and chromatin dynamics within specific tRNA domains and clusters illustrates that dynamic tDNA expression corresponds to changes in the surrounding environment. tRNA genes co-residing in clusters and contact domains within 200 Kb on chromosome 5, for example, exhibit similar transcriptional dynamics, both with nearby tRNA genes encoding distinct tRNA-types and with RNAPII-transcribed genes (Fig. 3a). A general decline in chromatin accessibility, H3K27 acetylation, and occupancy by RNA polymerase III is also observed across this locus, together suggesting that the topological organization of tDNAs within these physical contact domains may contribute to their expression and dynamic regulation during cellular differentiation.

### Coordinated long-range regulation of tRNA genes during cellular differentiation

**Fig. 4:**
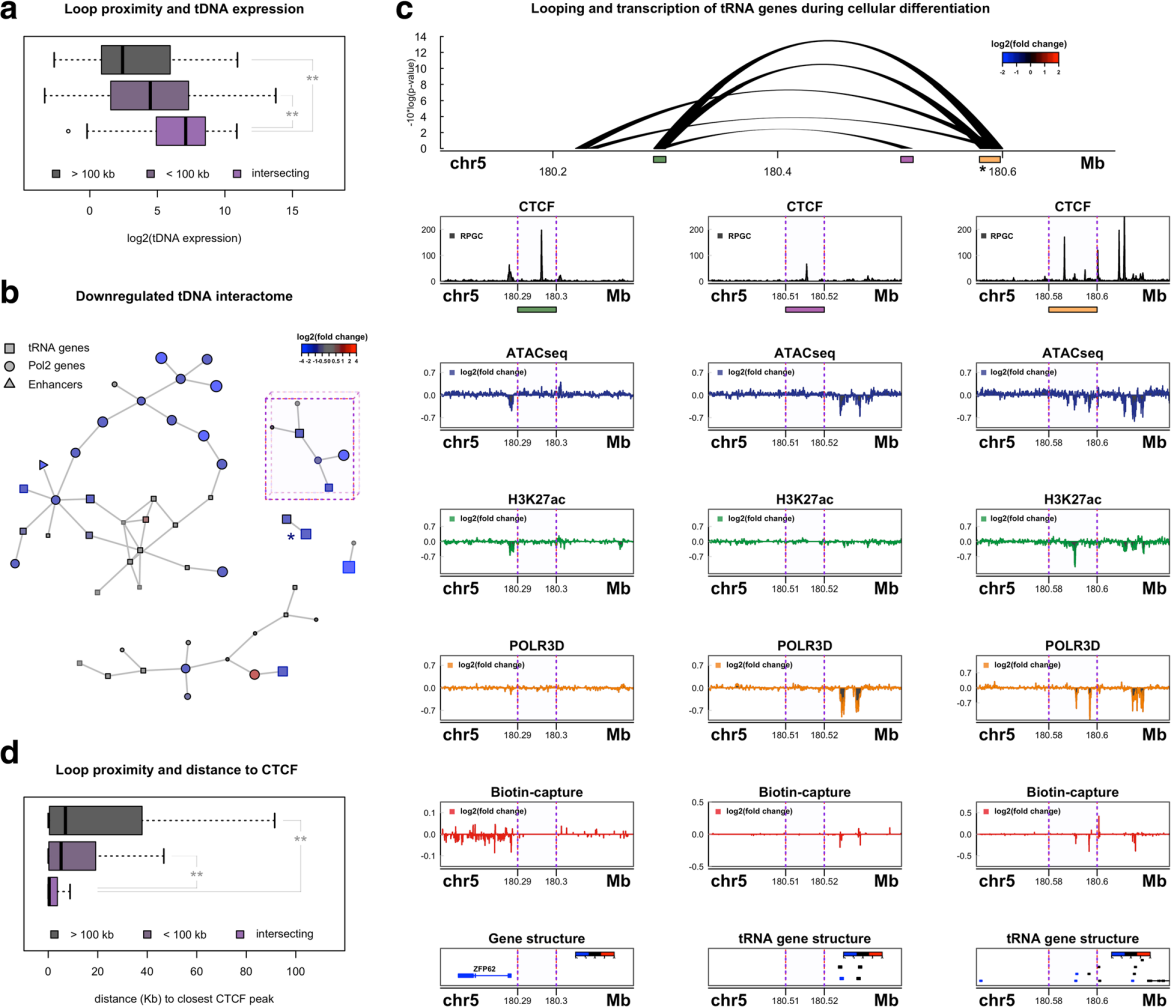
Coordinated long-range regulation of tRNA genes during cellular differentiation. **a** Distribution of integrated tDNA expression levels for tRNA genes > 100 Kb from a DNA loop end, within 100 Kb of a DNA loop end, and for tRNA genes that directly intersect DNA loop ends (*left* **p = 1.12^-6, *right* **p = 2.49^-11, Wilcoxon rank-sum test). **b** Network analysis of long-range interactions connecting tRNA genes downregulated in THP-1-derived macrophages. Each edge represents a DNA loop connecting two vertices (DNA loop anchors) that contain tRNA genes (*square*), RNAPII-transcribed genes (*circle*), or intergenic enhancers marked by H3K27 acetylation (*triangle*). *Vertices* with *black frames* represent loop anchors in which the identified feature (i.e. tRNA gene(s)) directly intersects the DNA loop end. *Vertices without black frames* represent loop anchors in which the identified feature is proximal to the DNA loop end (within 20 Kb). Both the size and color of each vertex is scaled by the mean log2(fold change) for resident feature(s). *Purple* outline marks the sub-community example further depicted in Fig. 4c. *Asterisk represents sub-community example further depicted in Additional file 1: Figure S5. **c** Visualization of chromatin and transcriptional dynamics at an example tDNA loop community located on chromosome 5. *Colored rectangles* define loop anchor regions further depicted below. *Asterisk represents loop anchor region depicted in Fig. 3a. *Bottom left*: signal track representation of CTCF binding sites (*black*, *RPGC* mean normalized reads per genomic content) and mean log2(fold change) for ATAC-seq (*blue*), H3K27ac (*green*), RNAPIII (*orange*), and nascent RNA (*red*) at the far-left loop anchor (*green rectangle*). Gene structure below includes RNAPII-transcribed gene *ZFP62. Vertical dotted lines* demarcate the actual loop anchor region. *Bottom middle*: analogous signal tracks depicting chromatin and transcriptional landscape at the middle loop anchor (*purple rectangle*) and proximal tDNA cluster. *Bottom right*: analogous signal tracks for the far-right loop anchor (*orange rectangle*) and intersecting tRNA genes. **d** Nearest distance to a CTCF binding site for tRNA genes that intersect DNA loop anchors, are within 100 Kb of a DNA loop anchor, or farther than 100 Kb from a DNA loop anchor (*left* **p = 2.31^-8, *right* **p = 6.61^-9, Wilcoxon rank-sum test)

In addition to interactions enriched within physical contact domains, Hi-C captures DNA loops connecting distant loci, ranging between several hundred kilobases (Kb) to megabase (Mb) in size [30]. More than 16,000 DNA loops were identified in THP-1 cells at 10-Kb resolution [45]. Most tDNAs are within 100 Kb of an identified loop anchor and nearly 20% of all tRNA genes are directly located at the end of a DNA loop (Additional file 1: Figure S4a–c). We find that transcription of tRNA genes is significantly higher for tDNAs directly intersecting loop anchors compared to tRNA genes within 100 Kb or tRNA genes that are located beyond 100 Kb from a loop end (Fig. 4a). These results suggest that the 3D organization of tRNA genes, which might include interactions that bring tRNA genes together in RNAPIII transcription factories [67], may also be important for regulating tDNA transcription during cellular differentiation.

We next asked what features are connected to tRNA genes by DNA loops and whether dynamic tDNA expression levels are coordinated by long-range interactions. Loop anchors identified by Hi-C often interact with more than one distant locus, forming multi-interaction networks or “hubs” that, in certain cases, connect multiple enhancers to target genes [45]. Analysis of all loop-associated downregulated tDNAs reveals a multi-interaction network that connects tRNA genes to other tDNAs as well as RNAPII-transcribed genes and intergenic enhancers marked by H3K27 acetylation (Fig. 4b). Features connected to tRNA genes by DNA loops show strikingly similar changes in transcription, suggesting tDNA dynamics are coordinated with other genes through long-range interactions during macrophage development (Fig. 4b). Visualization of individual tDNA interaction communities illustrates this phenomenon more clearly. For example, tRNA genes located at the boundary of adjacent tDNA contact domains on chromosome 5 (Fig. 3a) are directly located at a loop anchor that connects these tRNA genes to *ZFP62*, an RNA polymerase II-transcribed gene approximately 300 Kb upstream that concomitantly decreases in transcription in differentiating THP-1 cells (Fig. 4c, left and right signal track). DNA loops also bring *ZFP62* in close spatial proximity to additional tDNAs that decrease in transcription and RNA polymerase III occupancy (Fig. 4c, middle signal track), altogether illustrating coordinated long-range transcriptional downregulation after treatment with PMA.

In contrast to downregulated tRNA genes, we find that tDNAs that increase in transcription during cellular differentiation form interaction networks with comparatively few genes and with genes and regulatory elements that do not show significant changes after treatment with PMA. However, we find that nuclear-encoded mitochondrial tRNA genes, which generally increase in expression in developing macrophages, are proximal to DNA loops that connect genes and enhancers that behave similarly after differentiation, further reaffirming an important relationship between long-range interactions and coordinated dynamic transcription of human tRNA genes (Additional file 1: Figure S4f, g).

### Loop-based tRNA genes are connected by CTCF binding sites

Previous reports have identified enrichment for tRNA genes at the boundaries of topological domains, as well as a proximity relationship between specific tRNA genes and CTCF binding sites in eukaryotes [28, 62, 68–70]. Analysis of CTCF proximity with respect to tDNAs demonstrates that loop-associated tRNA genes are located near CTCF binding sites in THP-1 cells, consistent with recent models suggesting most long-range interactions are established by a loop extrusion complex that requires convergent CTCF binding sites (Fig. 4d) [36, 37, 40]. Inspection of DNA loops that bridge together tRNA genes clearly identifies CTCF binding at individual loop anchors, further suggesting that distal tRNA genes are brought together by CTCF (Fig. 4c). Supporting evidence for a functional role of CTCF in connecting tRNA genes is illustrated by an example in which two distinct clusters of tRNA genes, separated by more than 1.2 Mb on chromosome 6, interact via a DNA loop that is lost during macrophage differentiation, coincident with a loss of CTCF binding (Additional file 1: Figure S5). The tDNA clusters that are released by this long-range interaction are marked by decreasing tRNA gene transcription in THP-1 macrophages (Fig. 4b, asterisk), suggesting loss of DNA looping may be related to dynamic transcription regulation of tRNA genes.

Despite the observed relationship between tDNA transcription and CTCF-mediated long-range interactions, we do not identify any enrichment for differential tRNA genes at loop ends and, overall, changes in tDNA-associated long-range interactions are no more dynamic than non-tDNA loops (Additional file 1: Figure S4b, e). This suggests that while looping may be an important feature underlying tDNA expression levels, differentiation of THP-1 monocytes does not induce any widespread perturbation of the tDNA interactome in these cells. Indeed, regulation of tRNA genes is likely predominantly controlled by dynamic binding of TFs proximal to tDNAs within the framework of chromatin architecture. We therefore sought to further identify factors that might directly regulate tRNA gene expression during macrophage development.

### Downregulation of tRNA genes coincides with enhanced MAF1 occupancy

**Fig. 5:**
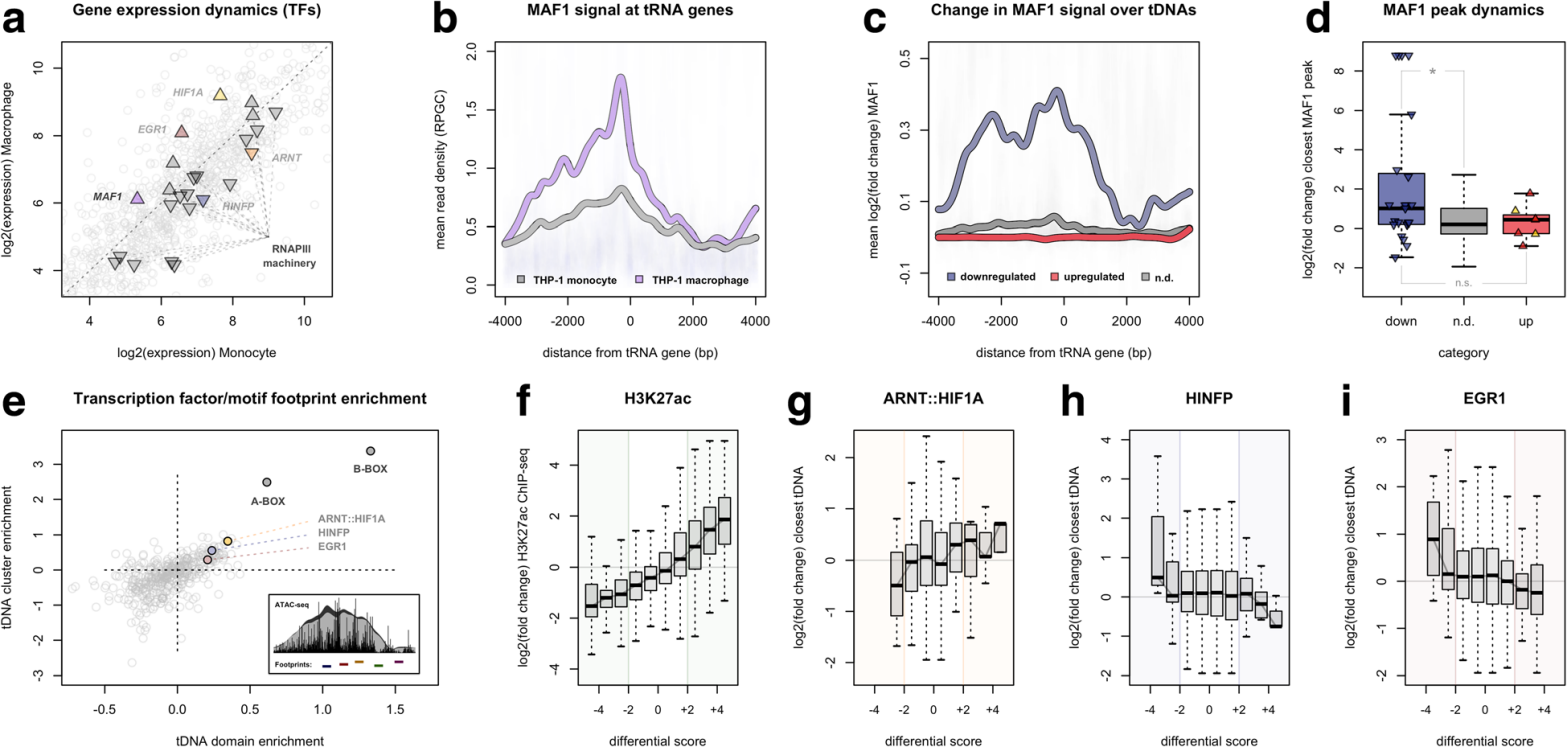
Differential transcription of tRNA genes coincides with enhanced MAF1 occupancy and dynamic TF binding. **a** Comparison of log2(expression) levels for TFs in THP-1 monocytes (*x-axis*) and THP-1 macrophages (*y-axis*). *Triangles* represent relevant genes encoding RNAPIII transcription machinery (*black*), *MAF1* (*purple*), *ARNT* (*orange*), *HIF1A* (*yellow*), *HINFP* (*blue*), and *EGR1* (*red*). *Triangles* do not denote statistical significance. **b** Mean read density of MAF1 ChIP-seq (*RPGC* mean normalized read per genomic content) at all tRNA genes in THP-1 cells before (*gray*) and after treatment with PMA (*purple*). **c** Mean log2(fold change) of MAF1 ChIP-seq read density at all downregulated (*blue*), upregulated (*red*), and non-differential (*gray*, n.d.) tRNA genes. **d** log2(fold change) of the closest MAF1 ChIP-seq peak for all downregulated (*blue*), upregulated (*red*), and non-differential (*gray*) tRNA genes (*p = 0.024, *n.s.* not statistically significant; Wilcoxon rank-sum test). **e** Enrichment of TF footprints identified by PIQ [75] within tDNA domains (*x-axis*) and clusters (*y-axis*). Enrichment measured as log10(observed/expected). *Inset*: illustration of ATAC-seq based footprinting analysis. Read ends represent hyperaccessible DNA adjacent to protected TF binding sites. **f** Comparison of dynamic H3K27ac levels at all footprints identified by PIQ. Differential accessibility scores were binned by the number of standard deviations from the average differential score across more than 2 million footprints (Additional file 1: Figure S6b). (+) denotes increased accessibility score, (−) denotes decreased accessibility score. *Colored overlay* represents strong differential accessibility score (≥2 standard deviations from the mean differential score) in THP-1-derived macrophages. **g** Comparison of dynamic ARNT::HIF1A footprint accessibility with the change in integrated tDNA expression at the nearest tDNA. **h** Comparison of dynamic HINFP footprint accessibility with the change in integrated tDNA expression at the nearest tDNA. **i** Comparison of dynamic EGR1 footprint accessibility with the change in integrated tDNA expression at the nearest tDNA

We find that the expression of TFs involved in RNA polymerase III initiation and transcription elongation moderately decreases after differentiation of THP-1 monocytes (Fig. 5a). This result is consistent with the downward bias for RNAPIII levels at tRNA genes after cellular differentiation and agrees with the expectation that tDNA regulation may be largely dictated through binding of TFIIIC and RNA polymerase III-related TFs. In contrast, nascent transcription of *MAF1*, a negative effector of RNA polymerase III activity, moderately increases after treatment with PMA (Fig. 5a). ChIP-seq experiments for MAF1 in monocytes and THP-1-derived macrophages confirm enhanced binding in the upstream promoter region for tRNA genes, particularly for tDNAs that decrease significantly after cellular differentiation (Fig. 5b, c). Downregulated tRNA genes also show significantly higher upregulation of MAF1 at the closest MAF1 peak (Fig. 5d, Additional file 1: Figure S6a), altogether consistent with the recently reported role of MAF1 in preventing RNA polymerase III recruitment and transcription initiation in humans [71]. The enhanced binding of MAF1 at a subset of tDNAs suggests that dynamic expression and binding of MAF1 is directed towards repressing specific tRNA genes during macrophage development and may therefore play an important role in controlling the dynamic range of nascent tRNA levels in humans. Collectively, these results agree with studies in yeast, in which deletion of the Maf1 transcriptional repressor results in differential sensitivity with respect to tRNA gene expression [72].

### Transcription factor footprinting uncovers candidate regulators of tDNA expression

The ends of read fragments generated by ATAC-seq can be used to identify regions of DNA that are directly bound by TFs and protected against fragmentation [73, 74]. To this end, we applied the Protein Interaction Quantification (PIQ) footprinting algorithm on ATAC-seq data generated in THP-1 cells, identifying in total more than 2 million footprints for 516 distinct TF motifs (Additional file 1: Figure S6b) [75, 76]. TF binding analysis identifies enrichment for specific regulatory elements within tDNA domains and tDNA clusters. As expected, both the A-BOX and B-BOX motifs, which represent internal tDNA regulatory elements bound by the TF complex TFIIIC [77], are substantially more enriched in tDNA clusters and tDNA contact domains than any other regulatory motif (Fig. 5e). Beyond TFIIIC binding sites, we also identify enrichment for several non-tDNA related regulatory elements. We therefore sought to further compare changes in TF binding during cellular differentiation with dynamic transcription of tRNA genes.

We observe a strong correlation between dynamic H3K27ac, a mark of active enhancers and promoters, with changes in chromatin accessibility over all TF footprints identified by PIQ (Fig. 5f) [78, 79]. ChIP-seq measurements of CTCF binding at intersecting motifs further validates the ability of this approach to capture dynamic binding of specific TFs (Additional file 1: Figure S6c). Analysis of dynamic chromatin accessibility for footprints enriched within tDNA domains and clusters uncovers strong correlations between TF occupancy and changes in nearby tRNA genes for specific motifs. For example, differential accessibility at ARNT::HIF1A footprints correlates with changes in tDNA transcription at proximal tRNA genes (Fig. 5g). Bound ARNT::HIF1A elements are strongly enriched in both tDNA clusters and contact domains, supporting a potential role for these factors in tDNA transcription regulation (Fig. 5e). We additionally observe correlations between tDNA transcription and footprint dynamics at HINFP and EGR1 regulatory elements (Fig. 5h, i), which are also enriched within tDNA clusters and domains. The expression of *HIF1A* and *EGR1*, both of which have been shown to significantly increase in PMA-treated THP-1 cells [80, 81], are also upregulated in our system after cellular differentiation (Fig. 5a), further validating the dynamic binding captured at these specific regulatory elements and supporting a possible role for these factors in dynamic tRNA gene regulation.

## Discussion

**Fig. 6:**
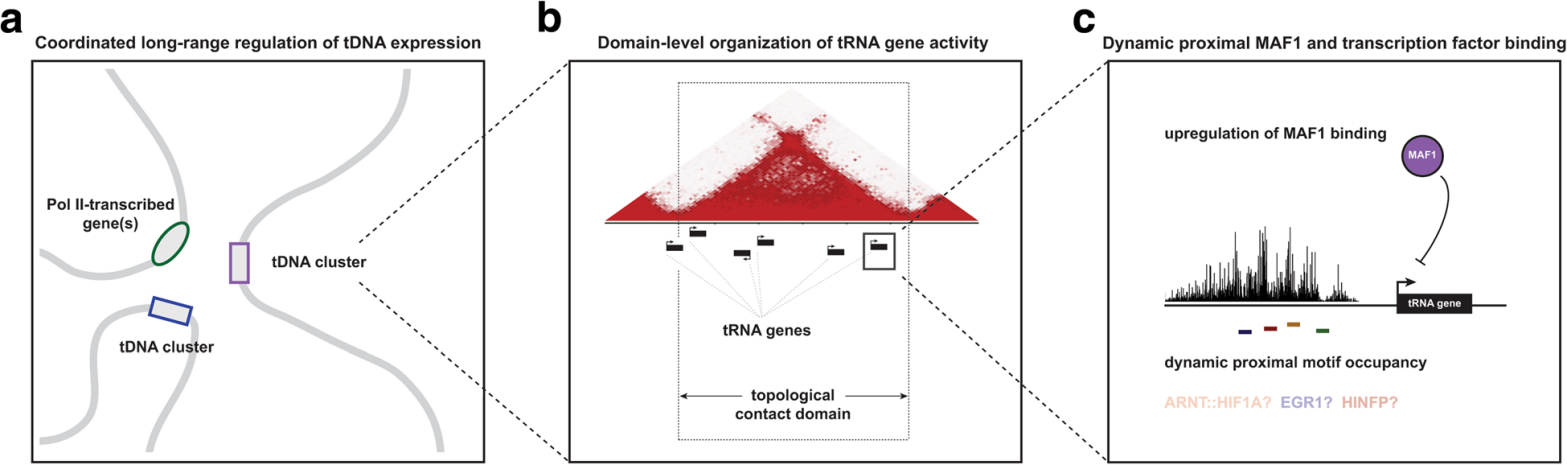
Model of multi-level tRNA gene regulation during macrophage development. **a** DNA loops bring together distal tRNA genes, RNAPII-transcribed genes, and enhancers that are coordinately downregulated during macrophage differentiation. **b** tDNAs are locally organized into clusters and physical contact domains that share similar activity and differential expression patterns in THP-1 cells. **c** Dynamic tDNA transcription correlates with proximal motif occupancy for specific, putative regulatory factors, such as HIF1A and EGR1. Downregulation of tRNA genes coincides with enhanced promoter-proximal binding of MAF1, a negative effector of RNAPIII activity

Integrated tDNA expression profiling uncovers dynamic transcription of individual tRNA genes during macrophage development in human THP-1 cells. By leveraging high-resolution maps of DNA interactions, chromatin accessibility, and histone and TF occupancy, we provide evidence of multi-level regulation of human tRNA genes during cellular differentiation, including long-range coordination of dynamic tDNA expression (Fig. 6a), cluster- and domain-level organization of tRNA gene activity (Fig. 6b), and canonical promoter-proximal regulation of individual tRNA genes (Fig. 6c). The systematic skew towards downregulation of most tRNA genes during monocyte-to-macrophage differentiation is consistent with previous RNA polymerase III mapping studies in other, distinct models of cellular differentiation. The repertoire of RNAPIII-bound and expressed tDNAs is more robust in human embryonic stem cells and induced pluripotent stem cells, for example, when compared to several differentiated cell types [56]. In the present study, we demonstrate for the first time that tDNA downregulation is organized across physical contact domains and through DNA loops connecting tRNA genes to distal tDNAs and RNAPII-transcribed genes. On the other hand, nuclear-encoded mitochondrial tDNAs are more likely to be upregulated and interact with genomic features that also increase after differentiation, a result that is congruous with an increasing number of mitochondria observed in THP-1-derived macrophages [48].

At the local level, downregulation of specific tRNA genes coincides with enhanced binding by MAF1, a transcriptional repressor that actively targets the promoter of RNAPIII genes [71, 82–88]. Though ectopic knock-down of *MAF1* in human IMR90 fibroblasts leads to enhanced RNAPIII occupancy at all expressed tRNA genes, we show that MAF1 shows preferential enrichment after THP-1 differentiation at specific, downregulated tDNAs, consistent with differential sensitivity of tRNA genes to Maf1 in yeast [71, 72]. TF footprinting analyses in THP-1 monocytes and macrophages also reveal enrichment for specific DNA motifs located near tRNA genes (Fig. 5e). Furthermore, dynamic binding at certain motifs correlates with differential tDNA expression patterns during differentiation, suggesting a potential role in tRNA gene activity. Regulatory elements targeted by ARNT::HIF1A, for example, are enriched within tDNA clusters and domains and binding dynamics at these elements correlate with tRNA gene dynamics in THP-1 cells. Hypoxic stress, which induces the accumulation and nuclear translocation of ARNT and HIF1A, has been shown to suppress RNAPIII recruitment and tDNA expression in cardiomyocytes and increase levels of tRNA-derived fragments in breast cancer and mammary epithelial cells [7, 89–91]. Thus, whether and to what degree these factors directly and actively influence the level of nascent cellular tRNAs remains an intriguing subject for future research [80].

Our study suggests that the 3D organization of the human genome is an important underlying feature of tRNA gene expression. A subset of tDNAs participates in long-range interactions and is expressed at significantly elevated levels compared to non-loop tDNAs. Importantly, tRNA genes that are proximal or intersect DNA loop anchors are strongly enriched for CTCF and inspection of several tDNA-associated interaction sites reveals that CTCF, rather than the tRNA genes themselves, are centrally enriched at DNA loop anchors. This result suggests that while tRNA genes and sites bound by the TFIIIC TF complex may contribute to long-range chromosome organization and subnuclear localization in eukaryotes, in humans, loop-based cis-interactions appear to be predominantly determined by CTCF and factors controlling cohesin association with DNA [35, 41, 42, 44, 92–97].

Overall, DNA loops bridging tRNA genes to distal tDNAs and RNAPII transcribed genes are relatively stable after PMA-induced differentiation. We find that downregulated tDNAs interact with genomic features that are similarly downregulated, suggesting tDNAs and non-tRNA genes are coordinately regulated within the framework of a stable chromatin architecture established by CTCF. This result is consistent with a minimal change in CTCF binding observed during THP-1 differentiation and the predominant role of static rather than dynamic loops in bridging dynamic enhancers to key regulatory genes during macrophage development [45]. Nevertheless, we do observe specific examples of dynamic tDNA-associated loops, such as a tDNA–tDNA interaction on chromosome 6 that is concomitantly lost and marked by decreasing tRNA gene expression during differentiation (Addition file 5: Figure S5). Loss of this long-range interaction coincides with diminished CTCF binding, reaffirming the important role of CTCF in establishing DNA loops connecting tRNA genes. This result is consistent with a recent manuscript suggesting that knockdown of either CTCF or cohesin subunit Rad21 leads to dynamic expression of human tRNA genes in mouse tail fibroblasts [98].

The dynamic expression of individual tRNA genes during cellular differentiation supports a more complicated structure governing nascent tRNA levels than simply tDNA copy number. Instead, differential transcription of human tRNA genes may provide a system for refining cellular tRNA pools towards cell-type-specific demands, consistent with recent examples of tRNA dynamics in response to specific perturbations [4, 5, 99]. In THP-1 cells, the decrease in nascent tRNA levels is likely harmonized with a slow-down in biosynthesis, as differentiation is accompanied by a loss in cellular proliferation and decreasing levels of regulatory kinases that control entry and progression through the cell cycle [100, 101]. It is worth noting that we do not observe any significant correlation between changes in the tRNA-type abundance and codon usage, as might be expected if tDNA dynamics were specially tuned toward cell-type-specific codon usage (Additional file 1: Figure S7a). This result is perhaps consistent with a recent report suggesting translational efficiency is stable across diverse cell types, regardless of cellular tRNA pools [102]. Instead, we find that decreasing tDNA expression most significantly diminishes the level of highly expressed tRNAs, a phenomenon that appears to be coordinated with a decrease in codon usage for the most frequently employed codons (Additional file 1: Figure S7b, c). We therefore speculate that the range of tDNA expression may be coupled to the dynamic range in mRNA levels and resulting codon usage frequencies in different cell types, rather than specific anticodon-codon optimization. While the present study addresses dynamics of tRNA synthesis at the transcriptional level, to what degree these changes impact the cellular levels of mature, aminoacyl-tRNAs available for translation remains an important question for future research.

## Conclusions

The transcription of tRNA genes, which are linearly organized into clusters and domains that share similar gene activity, generally decreases in developing macrophages, coincident with a decline in the dynamic range of transcriptomic codon usage. We find that downregulation of tRNA genes often occurs across topological domains and coordinately with other tRNA genes and RNA polymerase II-transcribed genes connected by DNA loops. We also show that MAF1, a negative effector of RNA polymerase III activity, increases at significantly downregulated tRNA genes during cellular differentiation, altogether revealing multiple levels of tDNA regulation during macrophage development.

## Methods

### THP-1 cell culture and differentiation

THP-1 cells were obtained from ATCC (Lot # 62454382) and grown for multiple passages in T-75 flasks of 2–8 × 10^5^ cells/mL in growth medium containing RPMI-1640 (Corning), 10% fetal bovine serum, and 1% penicillin streptomycin. For differentiation of THP-1 cells, non-adherent cells were diluted to 2 × 10^5^ cells/mL and grown overnight; a final concentration of 100 nM PMA was added the following morning. THP-1-derived macrophages were collected after 72-h exposure to PMA by aspirating media and any non-adherent cells and incubating adherent cells with TrypLE (ThermoFisher) for 15 min followed by cell wash in phosphate buffer saline (PBS) buffer.

### Chromatin immunoprecipitation (ChIP)

Equal numbers of THP-1 monocytes and THP-1-derived macrophages were collected (~10 million cells per ChIP experiment) and resuspended in growth media at 1 × 10^6^ cells/mL and cross-linked with rotation at room temperature in 1% formaldehyde for 10 min. Cross-linking was quenched with the addition of 200 mM glycine and an additional 5 min of rotation at room temperature. Cross-linked cells were then spun down and resuspended in 1× RIPA lysis buffer, followed by chromatin shearing via sonication (three cycles using a Branson sonicator: 30 s on, 60 s off; 15 additional cycles on a Bioruptor sonicator: 30 s on, 30 s off). Individual ChIP experiments were performed on pre-cleared chromatin using antibody-coupled Dynabead protein G (ThermoFisher) magnetic beads. Anti-histone H3 (acetyl K27) antibody was obtained from Abcam (ab4729), POLR3D antibody was obtained from abcam (ab86786; Lot# GR267691-1), MAF1 antibody was obtained from Santa Cruz Biotechnology (sc-365312 lot # G1411), and CTCF antibody was obtained from Millipore (07-729). A total of 3–5 ug of antibody per ChIP was coupled to 18-uL beads and rotated overnight with sheared chromatin at 4 °C. Beads were then washed 5× in ChIP wash buffer (Santa Cruz), 1× in TE, and chromatin eluted in TE + 1% SDS. Cross-linking was then reversed by incubation at 65 °C overnight, followed by digestion of RNA (30 min RNase incubation at 37 °C) and digestion of protein (30 min proteinase K incubation at 45 °C). ChIP DNA was then purified on a minElute column (Qiagen), followed by DNA library preparation and size selection of 350–550 bp fragments via gel extraction (Qiagen).

### Assay for transposase accessible chromatin (ATAC-seq)

Equal numbers of THP-1 monocytes and THP-1-derived macrophages were collected (50,000 cells per ATAC-seq experiment) and washed with 1× ice-cold PBS. Cells were pelleted via centrifugation (500 × g, 5 min, 4 °C) and resuspended in cell lysis buffer (10 mM Tris-HCl, pH 7.4, 10 mM NaCl, 3 mM MgCl_2_, 0.1% IGEPAL CA-630), and immediately spun down (500 × g, 10 min, 4 °C). The supernatant was then discarded, and transposition reaction carried out for 30 min at 37 °C with Tn5 transposase in transposition buffer (Illumina, cat#FC-121-1030). DNA was immediately purified on a minElute column (Qiagen), followed by PCR amplification using the NEBNext high-fidelity master mix (NEB cat#M0541) with nextera PCR primers and barcodes. PCR amplification was monitored as described [58], and gel purified to remove contaminating primer-dimer species.

### Biotin-capture of nascent RNA

Equal numbers of THP-1 monocytes and THP-1-derived macrophages were collected (~5 million cells per experiment) and washed 3× in ice-cold PBS, followed by resuspension and 5 min incubation in 10 mL ice-cold swelling buffer (10 mM Tris-Cl pH 7.5, 2 mM MgCl_2_, 3 mM CaCl_2_). Cells were pelleted at 4 °C and resuspended in 1 mL lysis buffer (swelling buffer + 0.5% Igepal, 10% glycerol, 2 u/mL SUPERase In (Ambion), and gently mixed 20× by pipetting with p1000 (pipette tip cutoff to reduce shearing). Nuclei were then pelleted (1000 × g) and washed once with lysis buffer, pelleted (1000 × g), and resuspended in 1 mL nuclear storage buffer (50 mM Tris-Cl pH 8.3, 40% glycerol, 5 mM MgCl_2_, 100 nM EDTA). Nuclei were again pelleted and resuspended in 100 uL nuclear storage buffer. Nuclei were mixed with an equal volume of run-on reaction buffer (10 mM Tris-Cl pH 8.0, 5 mM MgCl_2_, 1 mM DTT, 300 mM KCl, 20 u SUPERase In (Ambion), 1% Sarkosyl, + 0.375 mM biotin-11-C/UTP (Perkin-Elmer)) + 0.0375 mM biotin-11-A/GTP (Perkin-Elmer)), and incubated for 3 min at 30 °C. RNA was then extracted and isolated using the mirVana small RNA isolation kit (AM1560; Lot# 1412093). A total of 1 ug of purified small RNA was then incubated for 2 h at room temperature in 100 uL demethylation reaction buffer (600 mM KCl, 4 mM MgCl_2_, 100 uM NH_4_FeSO_4_, 600 uM α-ketoglutarate, 4 mM L-ascorbic acid, 100 ug/mL bovine serum albumin [BSA], and 100 mM MES buffer [pH 5]) with 80 pmol ALKB, 160 pmol ALKB D135S. Expression and purification of tag-free ALKB (Lot# 716634S01) and ALKB D135S (Lot# 711466S04) was carried out by GenScript (Piscataway, NJ, USA) and stored at ~ 0.2–0.5 mg/mL in 50 mM Tris-HCl, 150 mM NaCl, 10% Glycerol, pH 8.0. Demethylation experiments were quenched in 10 mM EDTA, followed by pull-down of nascent biotinylated RNA via streptavidin magnetic beads (NEB #S1420S). Beads were washed in high salt buffer (2 M NaCl, 50 mM Tris-Cl pH 7.4, 0.5% Triton X-100), medium salt buffer (300 mM NaCl, 10 mM Tris-Cl pH 7.4, 0.1% Triton X-100), and low salt buffer (5 mM Tris-Cl pH 7.4, 0.1% Triton X-100), and RNA library preparation was subsequently carried out on beads, using the NEBnext small RNA library preparation kit (NEB #E7330S/L) with the minor modifications that RNA was denatured at 80 °C prior to adaptor ligation, and reverse transcription was carried out at increasing temperatures (50 °C for 1 h, 60 °C for 30 min, 70 °C for 15 min). Following PCR amplification (12 cycles), DNA library was purified on a minElute column and subsequently size selected to remove primer dimer contamination.

### DNA sequencing and pre-processing

Biological replicates and experimental conditions (– PMA; + PMA) were sequenced together on an Illumina HiSeq2500 (paired-end, 100) for each individual experiment type (RNA-seq, ChIP-seq, biotin-capture) and sequencing reads trimmed using trim galore v. 0.4.0 (https://www.bioinformatics.babraham.ac.uk/projects/trim_galore/), before downstream sequence alignment and analyses.

### Integrated tDNA expression profiling

tRNA gene annotation and coordinates were collected from the genomic tRNA database (gtRNAdb) for *Homo sapiens* (hg19 – NCBI Build 37.1 Feb 2009) by downloading the tRNAscan-SE results bed file http://gtrnadb.ucsc.edu/genomes/eukaryota/Hsapi19/ [52, 53]. Mapping of tRNA fragments to tDNA coordinates nevertheless remains an imperfect process due to the multi-copy nature of many tRNA genes. Multiple alignment and analysis strategies have been proposed with distinct decision-making trees [50, 51]. For our specific interest in tDNA transcription profiling, we chose to map nascent RNA reads to the entire genome space to avoid false positives arising from sequence reads that are unrelated to tRNAs, which may occur when aligned to a limited reference set of tRNA genes [50]. In addition, the biotin-capture protocol ensures that tRNA fragments represent nascently transcribed RNA and thus special consideration of pre-tRNA and processed tRNA fragments was unnecessary. The presence of non-templated “CCA” at the 3′ terminus of mature tRNAs was not considered within the context of tRNA gene transcription. We further chose to limit multi-mapping reads to a single “best” alignment location to avoid erroneously increasing tDNA transcription estimation (as would occur with multi-mapping) or decreasing tDNA transcription estimation (as would occur if all multi-mapping reads were discarded).

Specifically, sequencing reads were filtered to a minimum size of 18 bp and the first sequencing read aligned to the hg19 reference genome with bowtie version 1.1.1 using options –k 1 “best” [103]. Aligned nascent RNA sequence reads were extracted over the coordinates of all tRNA genes, ± 100 bp, in addition to all annotated genes. Normalized read counts for each condition replicate were determined with the DESeq package for differential expression, using the estimateSizeFactors function on count datasets and counts function with option normalized = TRUE [104]. For integrated tDNA expression measurement, the mean normalized counts over tRNA genes, determined independently by biotin-capture RNA-seq and POLR3D ChIP-seq experiments, were taken for each condition and replicate. Integration of these two independent measures, which shows strong agreement (Fig. 1a), was chosen to benefit from the unique advantages of each assay. Importantly, inspection of integrated tDNA expression estimates demonstrates high correlation and reproducibility across biological replicate both before and after treatment with PMA (Additional file 1: Figure S1a). Nevertheless, in some cases, discrepancies in RNAPIII occupancy and nascent tRNA levels are observed for a subset of tDNAs (Fig. 1a). These differences potentially arise from differences in mappability, technical challenges specific to tRNA-seq, or to the indirect nature between POLR3D occupancy mapping, which alone may not equate to productive or efficient transcription of a given (tRNA) gene. We believe these differences give merit to the need for integrated tDNA expression profiling. Changes in tRNA gene expression levels before and after PMA treatment were determined as the mean fold change of integrated tDNA transcription across two biological replicates. tRNA genes that were considered downregulated and upregulated after 72 h PMA treatment show congruent changes in independent biotin-capture and RNAPIII mapping experiments with a false discovery rate (FDR) threshold of 0.15 (FDR determined using the Benjamini and Hochberg corrected exact test *p* value against integrated tDNA expression estimates).

### ChIP-seq analysis

Trimmed paired-end ChIP sequencing reads were mapped to the hg19 genome using bowtie version 1.1.1. with settings “bowtie –q –phred33-quals –X 2000 –fr –p 9 –S –chunkmbs 400” [103]. Mitochondrial reads were filtered and duplicate reads removed using Picard tools v. 1.92 (http://broadinstitute.github.io/picard). ChIP-seq peaks were identified for each condition using MACS2 v. 2.1.0 with options “macs2 callpeak –bdg –t –g hs” [105]. POLR3D ChIP-seq reads were extracted over tRNA genes, ± 100 bp, as well as all non-tRNA genes before normalization and integration with biotin-capture experiments for tDNA expression profiling. MAF1 binding dynamics were determined by differential count analysis over a merged MAF1 peak list using the exactTest function in the edgeR package for differential expression analysis [106, 107].

### ATAC-seq and TF footprinting analysis

Trimmed ATAC sequencing reads were mapped to the hg19 genome using Bowtie v 2.2.4 with settings “bowtie2 –t –sensitive” [108]. Mapped reads were merged across several sequencing replicates, before filtering mitochondrial reads and removing duplicate reads with Picard tools v. 1.92 (http://broadinstitute.github.io/picard). ATAC-seq peaks were identified for each condition using MACS2 v. 2.1.0 with options “macs2 callpeak –bdg –nomdel –t –g hs” [105]. Changes in chromatin accessibility were determined by differential read count analysis using the glmTreat function in edgeR over a merged list of peaks identified in each condition and biological replicate. TF footprinting on ATAC-seq data was broken into two steps: identifying bound TF motifs and determining the differential binding score at motifs bound in either or both conditions. Bound TF motifs were identified using the PIQ pipeline against motifs annotated in the Jaspar Core Vertebrate Database (http://jaspar.genereg.net) [75, 76]. Motif matches against the hg19 reference genome were identified using the PIQ package pwmmatch.exact.r script. TF footprint scores were then determined for each motif using the PIQ package pertf.bg.r and common.r scripts with default settings. Motifs that were considered bound were filtered at a minimum positive prediction value (PPV) of 0.7, as previously described for bound motif calling [75]. Differential TF binding scores for each motif were then determined using the Wellington-bootstrap algorithm for differential footprinting [78], using the bootstrap.py script for differential footprinting with command-line option “-A” for ATAC-seq input, followed by the pyDNase dnase_ddhs_scorer.py script for differential score calling over footprints identified by PIQ. Differential accessibility scores were median normalized and subsequently binned by standard deviation.

### Signal track and data visualization

Signal track data were generated from post-filtering read alignment bam files using the deeptools bamCompare tool [109, 110]. For individual sample conditions, normalized read per genomic count (RPGC) signal tracks were created by taking the mean ratio between biological replicates with bamCompare options “--ratio mean --normalizeTo1x 2451960000 --binSize 20 --smoothLength 60”. For ± PMA treatment comparison tracks, signal files were generated with bamCompare options “--pseudocount 30 --normalizeTo1x 2451960000 --binSize 20 --smoothLength 60”. Read density plots were generated using the deeptools computeMatrix tool with specified distances from strand-oriented tRNA genes [109, 110]. Signal track visualization and in situ Hi-C integration plots were generated using the Sushi package for genomic visualization [111]. Genome-wide tRNA gene circle visualization plots were generated using the RCircos package for Circos 2D track plots [112]. tDNA interactome network maps were generated using the R package iGraph for network analysis and visualization [113].

### tDNA cluster, domain, and loop calling

Clusters of tRNA genes were defined as a stretch of tRNA genes separated by a maximum tDNA-tDNA distance of 20 Kb. In other words, tDNAs within 20 Kb of another tRNA gene were grouped, with cluster size increasing until no remaining tRNA genes were within the specified distance. In situ Hi-C contact domains were defined in THP-1 monocytes using the previously described arrowhead algorithm at 5-Kb resolution with default Juicer parameters [30]. In total, 12,272 contact domains were identified [45]. tDNA domains were defined as any Hi-C contact domain, profiled in THP-1 cells by high-throughput chromosome conformation capture [45], that contains any tRNA gene(s). tRNA clusters and domains were scored by the number of resident tRNA genes. In some cases, contact domains are located within a larger overlapping contact domain. Thus, certain analyses avoid redundant tDNA-domain calling by assigning individual tRNA genes to the smallest resident tDNA contact domain. On the other hand, several tRNA genes are not within an identifiable contact domain and thus parallel analysis of tDNA clustering presents an analogous means of determining the role of proximal tDNA gene regulation. Loop-associated tRNA genes were defined as tRNA genes that intersect either end of a loop (10-Kb resolution), and comparisons of distance between tRNA genes and long-range interactions calculated as the shortest distance between individual tDNAs and loop ends (bedtools).

### Intra-cluster, intra-domain, and tDNA-interactome expression analyses

Intra-cluster range and IQR of integrated tDNA expression levels were determined for each unique tDNA cluster. tRNA genes were then randomly shuffled with respect to tDNA cluster assignment and the range and IQR permuted 100,000 times. Observed and expected ranges were compared across all clusters and domains (Additional file 1: Figure S1g) and with respect to cluster and domain size (Fig. 1e, f). In situ Hi-C contact domains containing tRNA genes were analyzed for intra-domain tDNA expression range and IQR in the same manner, with the exception that in cases where tRNA genes are present in multiple overlapping contact domains, these tDNAs were assigned to the single, smallest intersecting domain to avoid redundancy. tDNA interactome network analysis was generated using the iGraph R package for network analysis and visualization [113]. A graph object was created for all DNA loops mapped in THP-1 cells [45] with vertices representing loop anchors connected by edges (loops). All sub-network looping communities were detected using the fast greedy algorithm; communities that contain tRNA genes of interest (i.e. downregulated, upregulated, nmt-tDNA, etc.) were extracted for further analysis. All extracted community vertices were then characterized by intersecting or proximal features assigned by a ranking system: (1) intersecting tRNA genes; (2) proximal tRNA genes within four 10-Kb bins of a vertex; (3) non-tRNA genes that intersect a vertex; (4) non-tRNA genes that are proximal within two 10-Kb bins of a vertex; and (5) intergenic H3K27 acetylation peaks (putative enhancers) that directly intersect a vertex. Vertex shapes were determined by the highest ranked feature (tRNA genes = squares; non-tRNA genes = circles; putative enhancers = triangles), and the vertex shape and color scaled by the median log2(fold change) of the highest ranked feature(s).

### Calculation of median haploid tRNA gene copy number

We utilized a read depth approach to estimate the median tRNA gene copy number in humans. Specifically, whole-genome sequencing data for 15 individuals in the 1000 Genomes Project (six high-coverage genomes: NA12878, NA12891, NA12892, NA19240, NA19239, NA19238; nine moderate-coverage genomes: NA10847, NA12890, NA18489, NA18499, NA18504, NA18505, NA18519, NA19098, and NA19099) were mapped to the hg19 reference genome. Read coverage over individual tRNA genes were compared to 1000 randomly shuffled blocks of the same length to determine background coverage. Read coverage was then collapsed by anticodon tRNA families and tRNA gene count estimated by comparison to collapsed randomized background coverage (Additional file 1: Figure S2a).

## Additional file

Additional file 1: Figure S1. Domain and cluster-level organization of tDNA transcription (related to Fig. 1). **Figure S2.** tDNA copy number estimation and transcription in THP-1 cells (related to Fig. 2). **Figure S3.** Dynamic chromatin and tRNA transcription profiles during differentiation (related to Fig. 3). **Figure S4.** Dynamic chromatin and tDNA transcription profiles during differentiation (related to Fig. 4). **Figure S5.** Concurrent downregulation of tDNA looping and transcription (related to Fig. 4). **Figure S6.** MAF1 binding and differential TF footprinting during THP-1 differentiation (related to Fig. 5). **Figure S7.** Dynamic range of codon usage and aggregate tDNA expression levels during THP-1 differentiation. (PDF 8237 kb)
