# Supplementary material for: Topological organization and dynamic regulation of human tRNA genes during macrophage differentiation
Source: Genome Biol. 2017 Sep 20;18:180. doi: 10.1186/s13059-017-1310-3 (PMC5607496; doi:10.1186/s13059-017-1310-3)
Supplement: Additional file 1: Figure S1. — Domain and cluster-level organization of tDNA transcription (related to Fig. 1). Figure S2. tDNA copy number estimation and transcription in THP-1 cells (related to Fig. 2). Figure S3. Dynamic chromatin and tRNA transcription profiles during differentiation (related to Fig. 3). Figure S4. Dynamic chromatin and tDNA transcription profiles during differentiation (related to Fig. 4). Figure S5. Concurrent downregulation of tDNA looping and transcription (related to Fig. 4). Figure S6. MAF1 binding and differential TF footprinting during THP-1 differentiation (related to Fig. 5). Figure S7. Dynamic range of codon usage and aggregate tDNA expression levels during THP-1 differentiation. (PDF 8237 kb) [file 13059_2017_1310_MOESM1_ESM.pdf]

# Additional File 1:

## Supplemental Figures 1 – 7

Topological organization and dynamic regulation of human tRNA genes during macrophage differentiation

Kevin Van Bortle[1], Douglas H. Phanstiel[2,3], and Michael P. Snyder[1]

[1]Department of Genetics, Stanford University, Stanford, CA 94305, USA.,

[2] Department of Cell Biology and Physiology, [3]Thurston Arthritis Research Center and Department of Cell Biology and Physiology, University of North Carolina, Chapel Hill, NC 27599, USA.

**Running title:** Multi-level control of tRNA gene expression during cellular differentiation

# Supplemental Figure 1

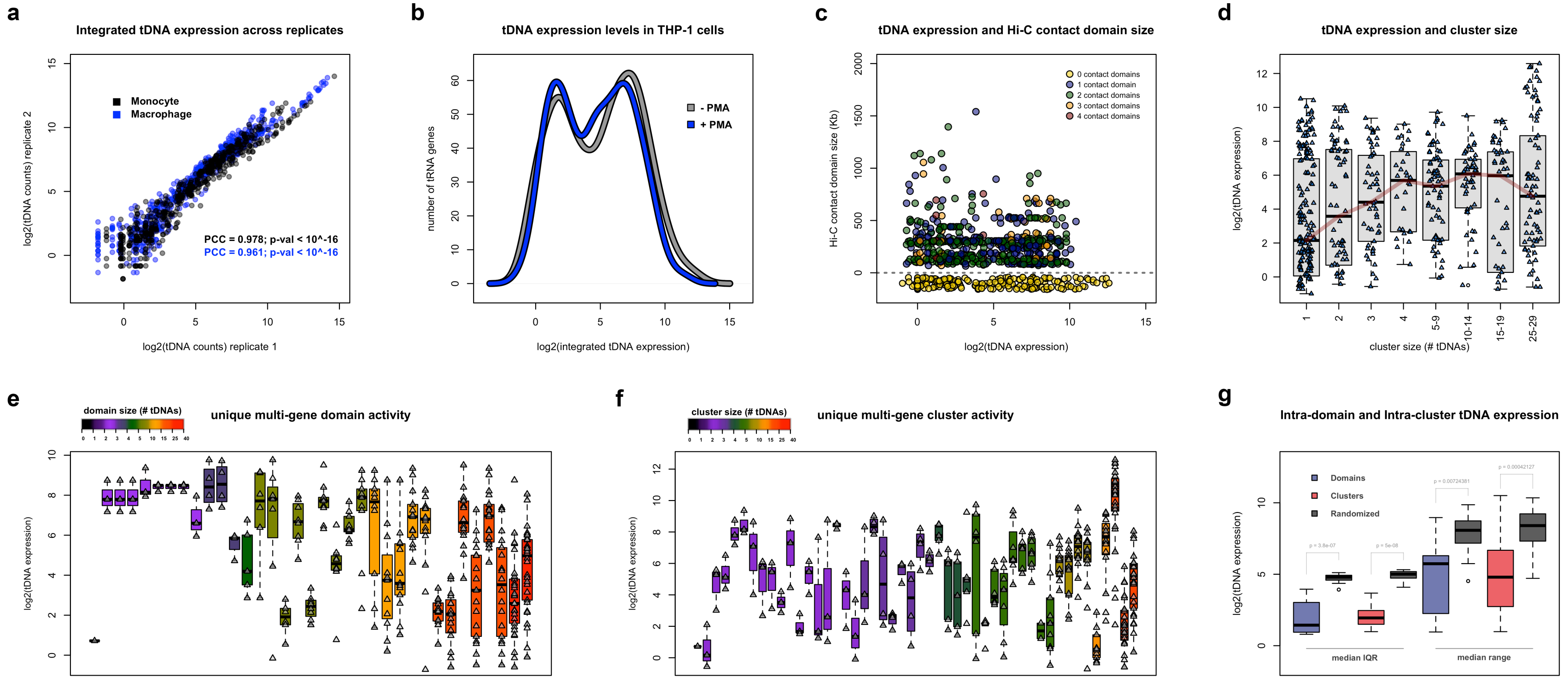


**Figure S1. Domain and cluster-level organization of tDNA transcription (related to Figure 1).** (**a**) Correlation comparison of integrated tDNA expression estimates across biological replicates in THP-1 monocytes (black) and THP-1 derived macrophages (blue). PCC = Pearson correlation coefficient (**b**) Distribution of integrated tRNA gene expression levels in THP-1 monocytes (black) and THP-1 derived macrophages (blue). (**c**) log2(integrated tDNA expression) with respect to resident contact domain size (Kb = kilobase). Color represents contact domain membership for individual tRNA genes in terms of number of overlapping physical contact domains. (**d**) log2(integrated tDNA expression) with respect to the size of each tRNA gene's cluster membership (size = total # of cluster tRNA genes). (**e**) log2(integrated tDNA expression) across individual multi-tDNA contact domains, ordered and colored by the domain size (# tRNA genes). Each boxplot represents a unique domain and the distribution of transcription levels across all resident tRNA genes (triangles). (**f**) log2(integrated tDNA expression) across individual tDNA clusters. Clusters defined by proximity (maximum tDNA-tDNA distance of 20 Kb). Each boxplot represents a unique multi-tDNA cluster and the distribution of transcription levels across all resident tRNA genes (triangles). (**g**) Range and interquartile range of log2(integrated tDNA expression) across all tDNA clusters (red) and all contact domains (blue) compared to randomized tDNA occupancy (black). P-values determined by Wilcoxon rank-sum test.

# Supplemental Figure 2

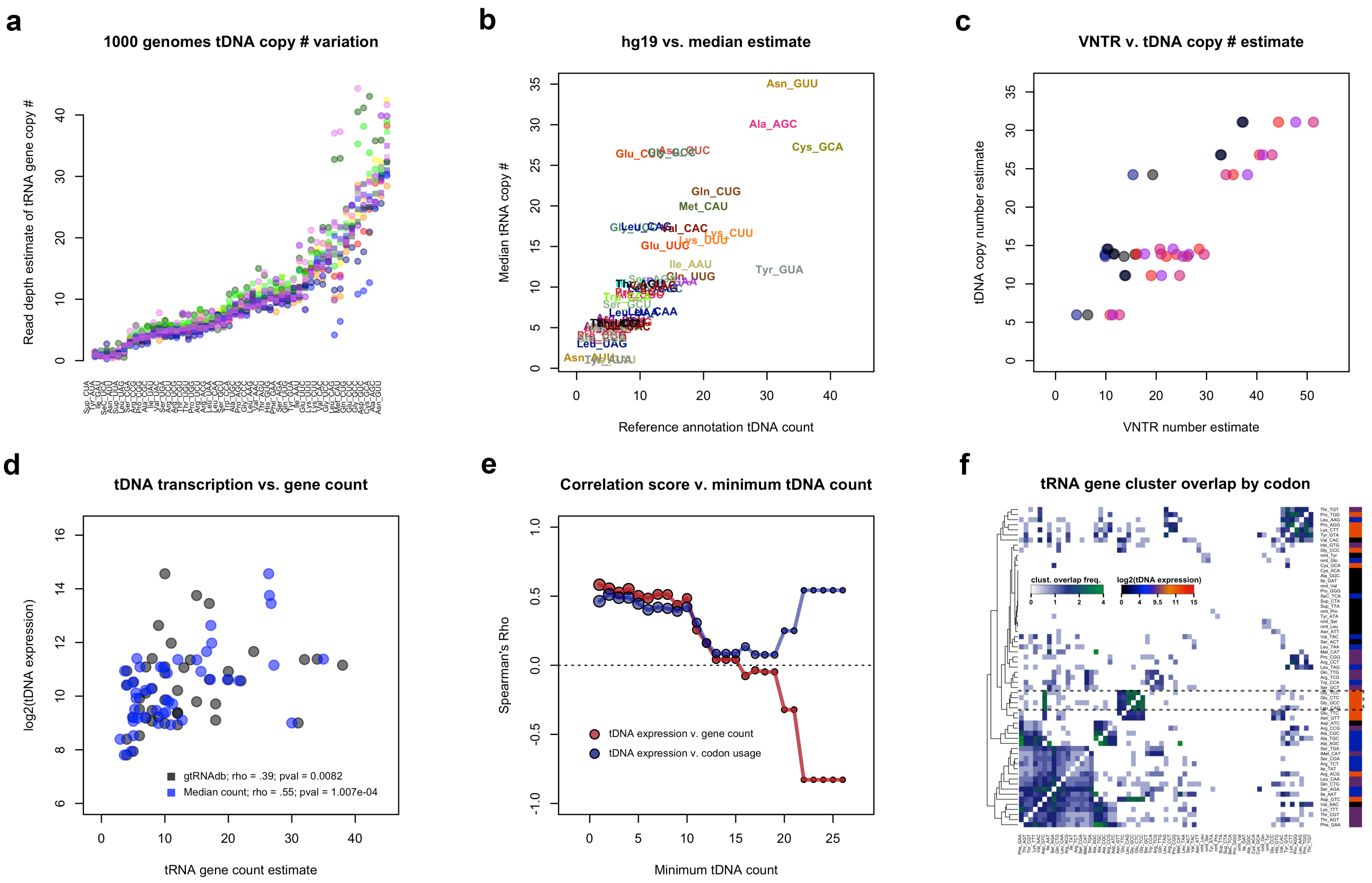


**Figure S2. tDNA copy number estimation and transcription in THP-1 cells (related to Figure 2).** (**a**) Visualization of tRNA gene copy number estimates determined using a read-depth measurement (compared to randomly shuffled background coverage) across several publicly available 1000 Genomes Project whole genome sequence datasets. (square = high coverage WGS; circle = moderate coverage WGS). (**b**) Correlation between genomic tRNA database (hg19) annotated tRNA gene count versus the median estimated

tRNA gene copy number determined across 1000 Genomes Project samples. (**c**) Comparison of estimated tRNA gene copy number at high variation tRNA genes with estimated variable number tandem repeat (VNTR) across several individuals (Leu_CAG = black; Clu_CUC = red; Gly_GCC = purple; Asp_GUC = pink; Gly_UCC = blue). (**d**) Comparative correlation analysis between integrated tDNA expression estimates in THP-1 monocytes with estimated tRNA gene copy number (black = gtRNAdb; blue = median tRNA gene copy number estimated here). Rho = Spearman's Rank Correlation Coefficient. (**e**) Correlation estimates between integrated tDNA expression levels with median estimated gene count (red) and codon usage in the THP-1 transcriptome (blue). X-axis represents the minimum tDNA copy number filtered before correlation estimate. Correlations diverge significantly when exclusively considering high copy number tDNAs. (**f**) Hierarchical clustering (Manhattan distance; complete linkage) of overlap frequency between anticodon tRNA family within tDNA clusters. That is, the number of times two genes encoding distinct anticodon tRNA families are members of the same tDNA cluster. (freq = fraction of all overlaps). Right-side colorbar represents the aggregate integrated tDNA expression levels for each anticodon tRNA family.

## Supplemental Figure 3

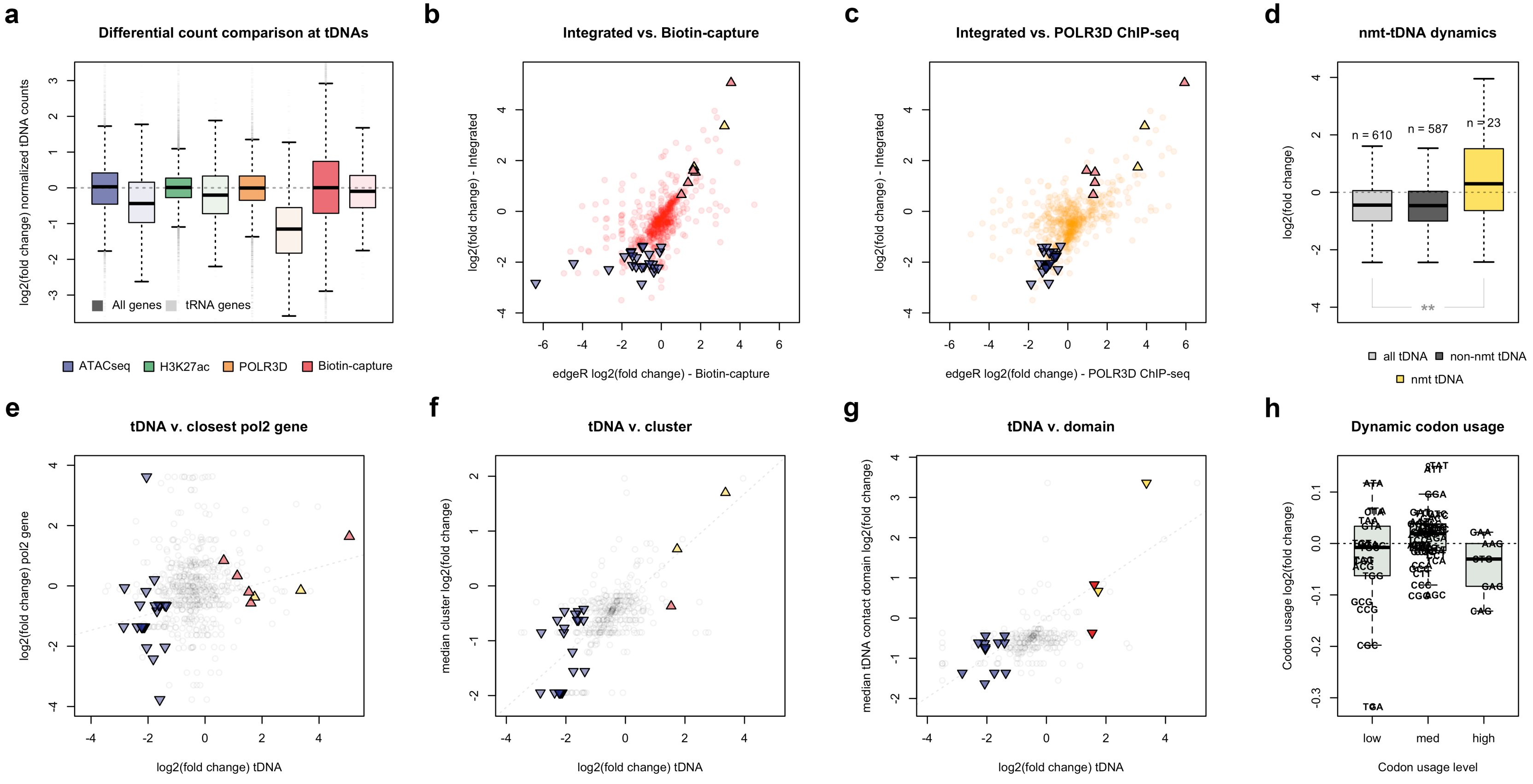


**Figure S3. Dynamic chromatin and tRNA transcription profiles during differentiation (related to Figure 3).** (**a**) Change in chromatin accessibility (ATAC-seq, blue), H3K27ac levels (ChIP-seq, green), RNA polymerase III occupancy (POLR3D ChIP-seq, orange), and nascent RNA levels (Biotin-capture, red) across all genes (dark shade) and across tRNA genes (light shade). (**b**) Correlation between log2(fold change) for integrated tDNA expression estimates (y-axis) with the change in nascent tRNA levels (Biotin-capture, x-axis). Triangles represent downregulated (blue) and upregulated tDNAs (red; nuclear encoded mitochondrial tRNA genes (nmt-tDNAs) gold) filtered at a maximum FDR cutoff of 0.15. (**c**) Analogous correlation between log2(fold change) for integrated tDNA expression estimates (y-axis) with the change in RNAPIII occupancy (POLR3D ChIP-seq, x-axis). (**d**) Comparison of integrated tDNA expression changes for all tRNA genes (gray), non-nmt-tDNAs (gray), and nmt-tDNAs (gold) in THP-1 derived macrophages. (* p = 0.0239, Wilcoxon rank-sum test). (**e**) Correlation analysis comparing integrated differential tDNA transcription with changes in RNA polymerase II transcription at the nearest gene (related to Figure 3E). (**f**) Correlation analysis comparing integrated differential tDNA transcription with the median intra-cluster change of tDNAs across the same cluster for each individual tRNA gene (related to Figure 3F). (**g**) Correlation analysis comparing integrated differential tDNA transcription with the median intra-

domain change of tDNAs across the same physical contact domain for each individual tRNA gene (related to Figure 3G). (**h**) Boxplot distributions of dynamic codon usage in THP-1 monocytes and THP-1 derived macrophages for low (< 0.01), medium (0.02-0.03), and high usage codons (> 0.03). Codon usage was calculated for each gene and corrected for transcript abundance. The mean codon usage (x-axis) was determined across replicates, and represents the fraction of all codons that an individual triplet represents. The log2(fold change) (y-axis) represents the change in codon usage after differentiation.

## Supplemental Figure 4

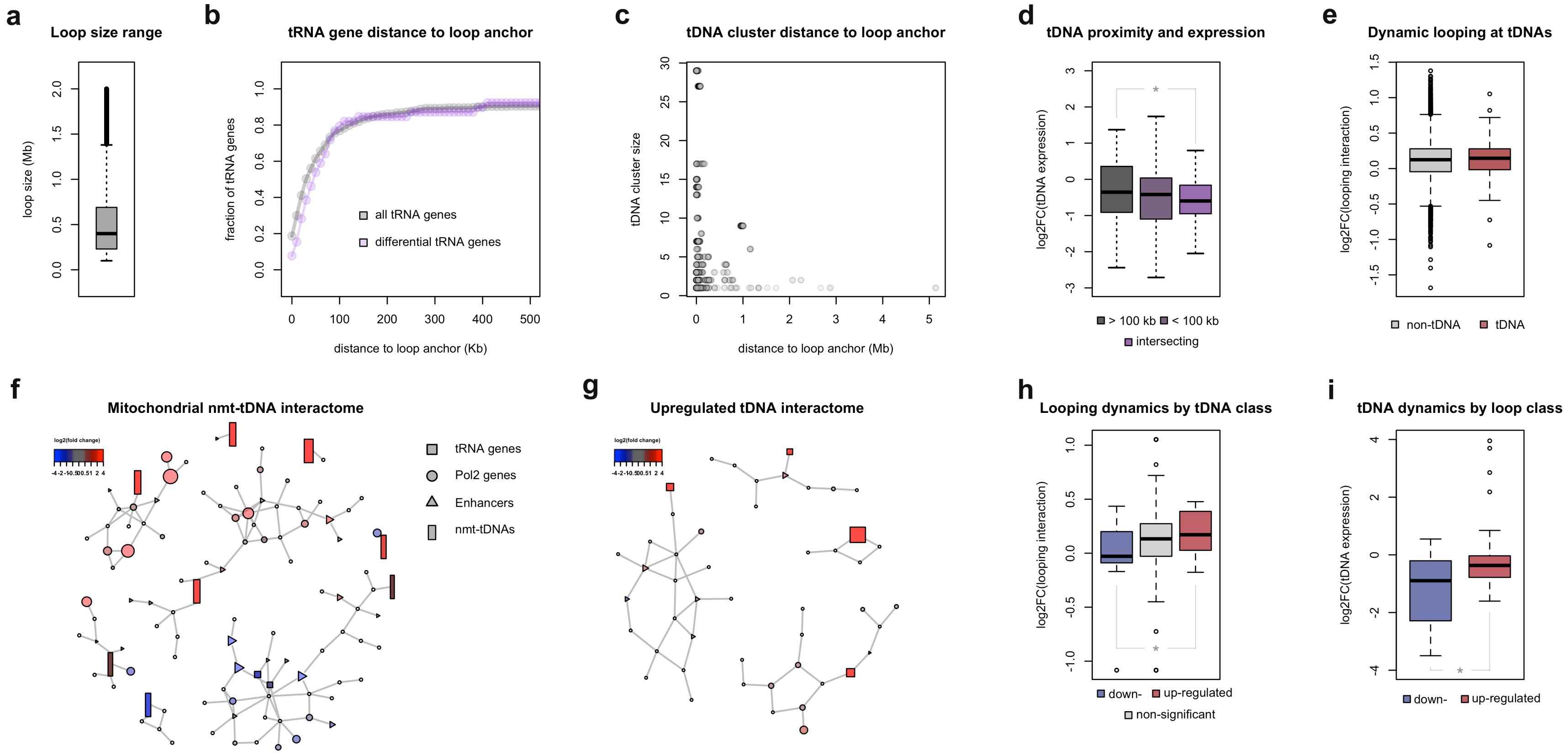


**Figure S4. Dynamic chromatin and tDNA transcription profiles during differentiation (related to Figure 4).** (**a**) Size distribution of long-range interactions mapped by in situ Hi-C in human THP-1 cells at 10 Kb resolution. (**b**) Fraction of tRNA genes within specified distance to loop ends (Kb), for all tRNA genes (black), and for tRNA genes defined as increasing or decreasing after differentiation (pink). (**c**) Distance between each individual tRNA gene, represented by resident tRNA cluster size, and loop end (Mb). (**d**) Distribution of log2(fold change) in integrated tDNA expression levels for genes at loop ends, within 100 Kb, or greater than 100 Kb from a DNA loop end (* $p = 0.0178$, Wilcoxon rank-sum test). (**e**) Distribution of log2(fold change) in long-range interaction contact frequencies across all non-tDNA loops (gray) and tDNA-associated DNA loops (red). (**f**) Network analysis of long-range interactions connecting nuclear encoded mitochondrial tRNA genes (nmt-tDNAs) in THP-1 derived macrophages. Each edge represents a DNA loop connecting two vertices (DNA loop anchors) that contain nmt-tDNAs (rectangle), tRNA genes (square), RNAPII-transcribed genes (circle), or intergenic enhancers marked by H3K27 acetylation (triangle). Both the size and color of each

vertex is scaled by its mean log2(fold change) for resident feature(s). (**g**) Analogous network analysis of long-range interactions that connect non-nmt-tDNAs that are upregulated in THP-1 derived macrophages. (**h**) Distribution of log2(fold change) in long-range interactions at tRNA genes that decrease (blue), increase (red), or do not change significantly (gray) after differentiation (* $p = 0.0170$, Wilcoxon rank-sum test). (**E**) log2(fold change) in integrated tDNA expression levels for genes proximal or intersecting DNA loops that significantly decrease (blue) or increase (red) after macrophage differentiation (* $p = 0.045$, Wilcoxon rank-sum test).

# Supplemental Figure 5

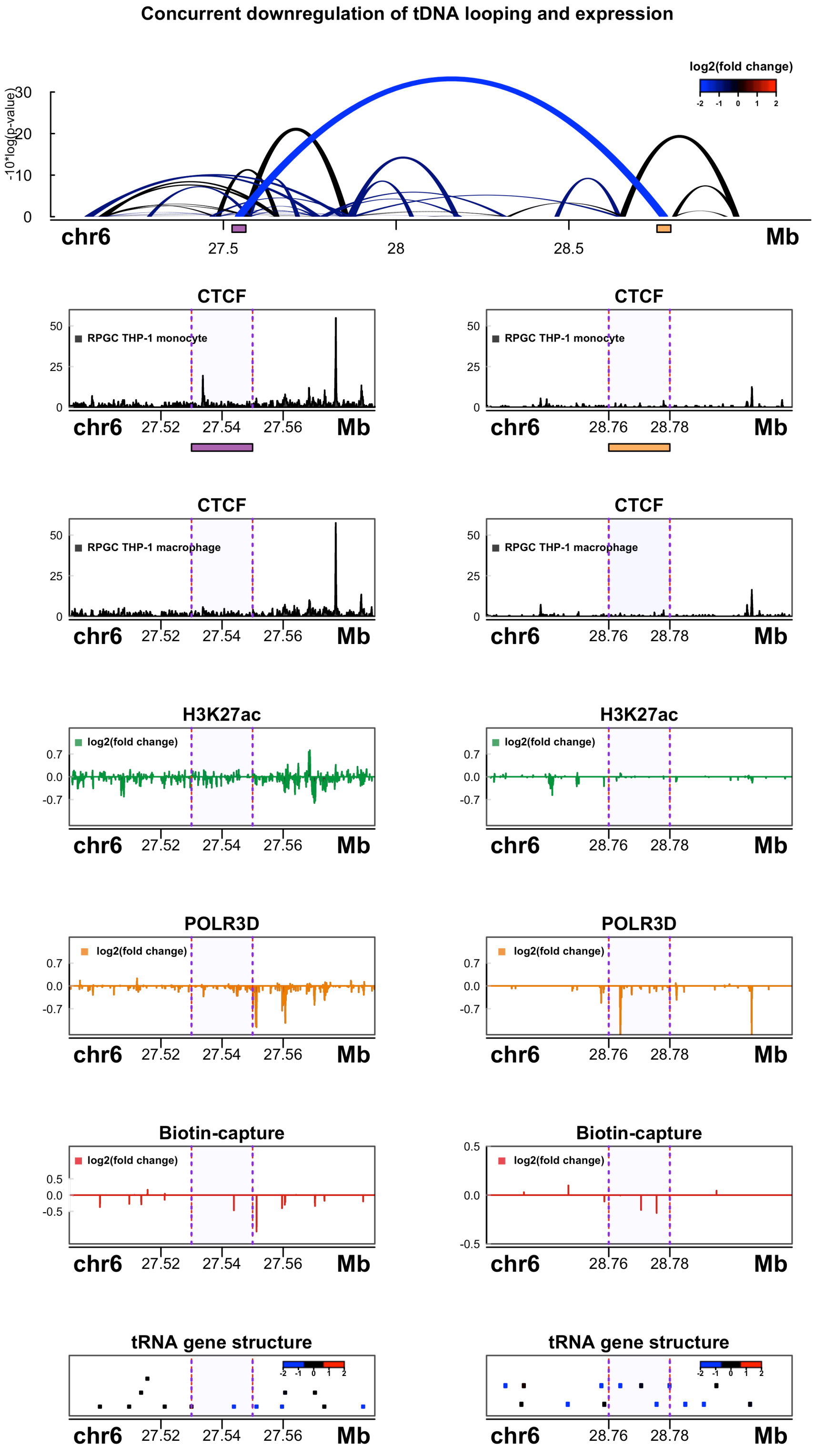


**Figure S5. Concurrent down-regulation of tDNA looping and transcription (related to Figure 4).** Examination of chromatin and RNA profiles at either end of a 1.2 Mb tDNA-tDNA loop on chromosome 6 that significantly decreases during macrophage development. Colored rectangles define loop anchor regions further depicted below. Bottom Left: Signal track representation of CTCF binding sites (black, RPGC = mean normalized reads per genomic content) before and after 72 hour PMA treatment, and mean log2(fold change) for H3K27ac (green), RNAPIII (orange), and nascent RNA (red) at the far-left loop anchor (purple rectangle) and intersecting / proximal tDNA cluster. Vertical dotted lines demarcate the actual loop anchor region. Bottom Right: Analogous signal tracks depicting chromatin and transcriptional landscape at the far-right loop anchor (orange rectangle) and intersecting /proximal tDNA cluster.

# Supplemental Figure 6

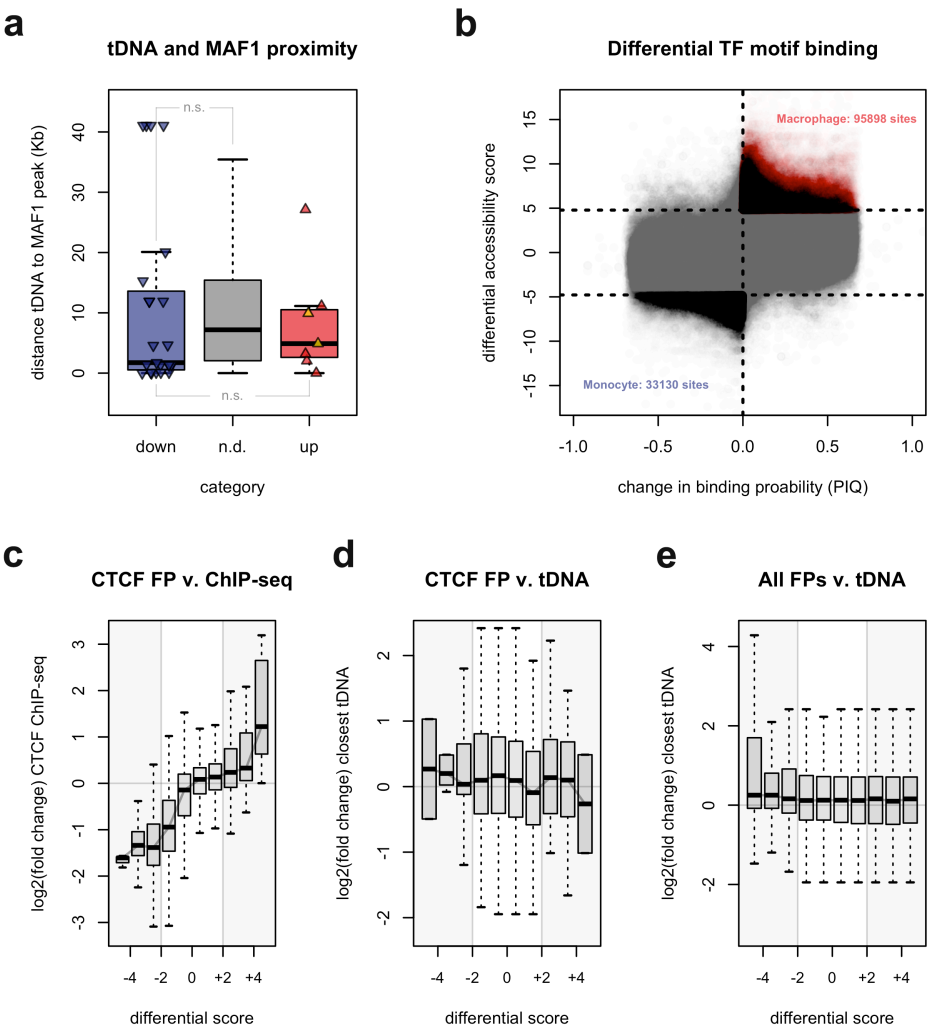


**Figure S6. MAF1 binding and differential transcription factor footprinting during THP-1 differentiation (related to Figure 5).** (**a**) Comparison of distance (Kb) between tRNA genes and the closest identified MAF1 ChIP-seq peak for tDNAs that decrease (blue), increase (red), or do not change significantly (gray) after differentiation. (n.s. = not statistically significant). (**b**) Comparison of differential accessibility scores at motifs matched by Protein Interaction Quantification (PIQ) across the hg19 reference genome, using the Jaspar Core Vertebrate motif database (http://jaspar.genereg.net). 2,731,616 footprints with a positive predicting value (PPV) cutoff of 0.7 were mapped against 516 distinct TF motifs (median 3,693 binding sites per motif). Differential accessibility scores (y-axis) were compared against changes in positive predicting value (PPV) as determined by PIQ (x-axis). Significantly differential binding events were arbitrarily defined as any footprint greater than +/- two standard deviations from the median differential accessibility score, with matching directionality in binding probability as determined by PIQ. At this threshold, a total of 33,130 TF binding sites significantly decrease 72 hours post PMA treatment (blue), whereas 95,898 TF binding sites increase (red). (**c**) Change in CTCF footprint accessibility score (x-axis) binned by standard deviation versus the change in CTCF binding at overlapping peak. (**d**) Change in CTCF footprint accessibility score at the nearest, non-overlapping regulatory element, versus change in tRNA gene transcription. (**e**) Relationship between differential TF binding and changes in integrated tRNA gene transcription for all identified regulatory motifs.

## Supplemental Figure 7

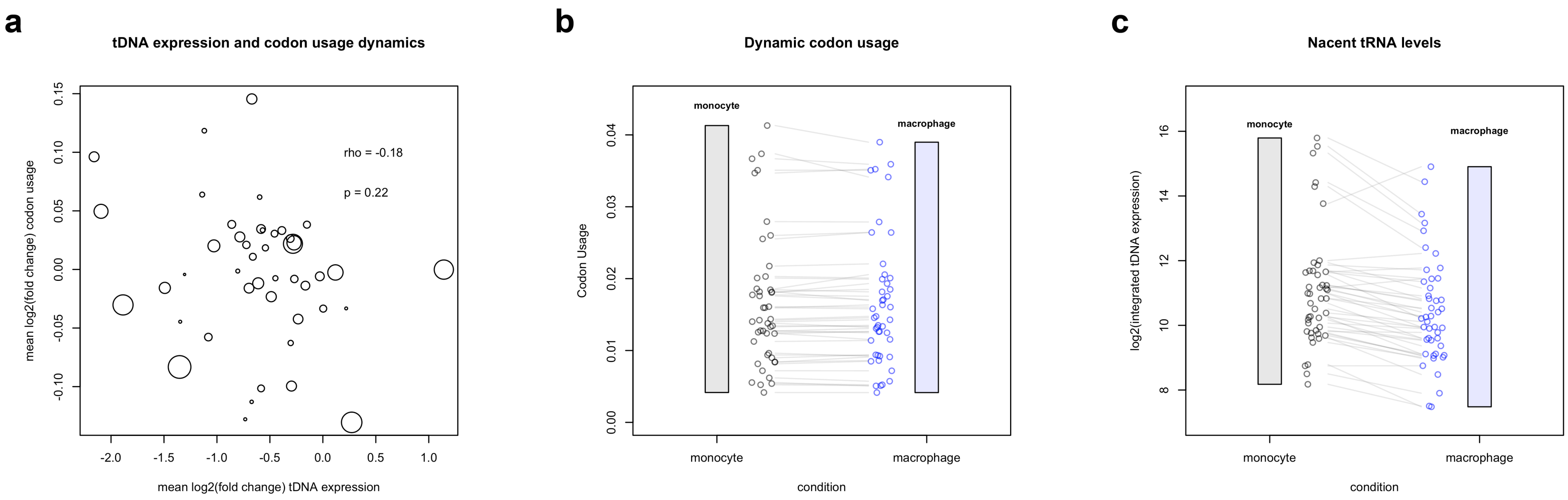


**Figure S7. Dynamic range of codon usage and aggregate tDNA expression levels during THP-1 differentiation.** (**a**) Correlation between aggregate log2(fold change) of tDNA transcription levels (x-axis) and the log2(fold change) in codon usage frequency before and after 72 hour PMA treatment. Circle diameter is scaled by average codon usage in THP-1 cells. (**b**) Upper and lower limits for codon usage in the transcriptomes of THP-1 monocytes and THP-1 derived macrophages. Line segments connect individual codons between conditions. (**c**) Analogous upper and lower limits of aggregate tRNA levels based on integrated tDNA expression profiles before and after PMA treatment.
